# Contextual modulation of value signals in reward and punishment learning

**DOI:** 10.1038/ncomms9096

**Published:** 2015-08-25

**Authors:** Stefano Palminteri, Mehdi Khamassi, Mateus Joffily, Giorgio Coricelli

**Affiliations:** 1 Institute of Cognitive Neuroscience (ICN), University College London (UCL), London WC1N 3AR, UK; 2 Laboratoire de Neurosciences Cognitives (LNC), Département d’Etudes Cognitives (DEC), Institut National de la Santé et Recherche Médical (INSERM) U960, École Normale Supérieure (ENS), 75005 Paris, France; 3 Instintut des Systèmes Intelligents et Robotique (ISIR), Centre National de la Recherche Scientifique (CNRS) UMR 7222, Université Pierre et Marie Curie (UPMC), 70013 Paris, France; 4 Interdepartmental Centre for Mind/Brain Sciences (CIMeC), Università degli study di Trento, 38060 Trento, Italy; 5 Groupe d’Analyse et de Théorie Economique, Centre National de la Recherche Scientifique (CNRS) UMR 5229, Université de Lyon, 69003 Lyon, France; 6 Department of Economics, University of Southern California (USC), 90089-0253 Los Angeles, California, USA

## Abstract

Compared with reward seeking, punishment avoidance learning is less clearly understood at both the computational and neurobiological levels. Here we demonstrate, using computational modelling and fMRI in humans, that learning option values in a relative—context-dependent—scale offers a simple computational solution for avoidance learning. The context (or state) value sets the reference point to which an outcome should be compared before updating the option value. Consequently, in contexts with an overall negative expected value, successful punishment avoidance acquires a positive value, thus reinforcing the response. As revealed by post-learning assessment of options values, contextual influences are enhanced when subjects are informed about the result of the forgone alternative (counterfactual information). This is mirrored at the neural level by a shift in negative outcome encoding from the anterior insula to the ventral striatum, suggesting that value contextualization also limits the need to mobilize an opponent punishment learning system.

## Abstract (web summary)

In contrast to predictions from learning theory, humans learn to seek rewards and avoid punishments equally well. Here the authors offer an elegant solution to this problem by demonstrating that humans learn option values relative to a reference point subserved by a common neural substrate.

## Graphical abstract

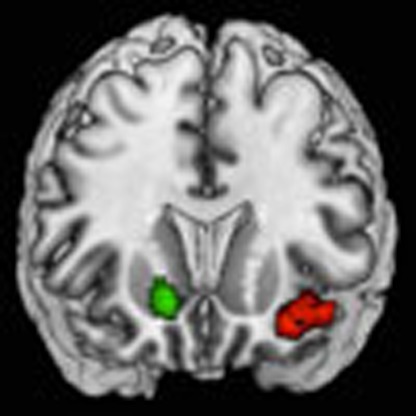

In the past decades, significant advances have been made in the understanding of the computational and neural bases of reward-based learning and decision making. On the other hand, computations and neural mechanisms mediating punishment-based learning and decision making remain more elusive^1^,^2^.

The first problem is computational. In fact, avoidance learning faces an apparent paradox: once a punishment is successfully avoided, the instrumental response is no longer reinforced. As a consequence, basic learning models predict better performance on reward learning (in which the extrinsic reinforcements are frequent, because they are sought) compared with punishment learning (in which the extrinsic reinforcements are infrequent, because they are avoided), despite the fact that human subjects have been shown to learn equally well in both domains^3^,^4^,^5^,^6^.

The second problem is neuroanatomical: a debate in cognitive neuroscience concerns whether the same brain areas (namely the ventral striatum and the ventromedial prefrontal cortex) represent positive as well as negative values or, alternatively, aversive value encoding and learning are organized in an opponent system (namely the insula and the dorsomedial prefrontal cortex)^7^,^8^,^9^,^10^,^11^,^12^.

We hypothesized that the two questions could be resolved in the framework of value context dependence. Recently, context dependence of option values has provided a formal framework to understand adaptive coding and range adaptation of value-responsive neurons and brain areas^13^,^14^,^15^,^16^. Concerning punishment learning, operationalizing the principle behind the two-factor theory, we propose that successful avoidance, which is a neutral outcome in an absolute scale, acquires a positive value because it is computed relative to the value of its choice context, which is negative^17^,^18^,^19^. In other words, successful avoidance is ‘reframed’ as a positive outcome^20^. On the other side, divergent functional magnetic resonance imagining (fMRI) findings could be reconciled assuming that, in absence or limited contextual information, punishments and rewards are implemented in opponent channels; subsequently, if contextual information is acquired or provided, outcome representations converge to the ventral frontostriatal system. This is supported by the fact that ventral striatal and prefrontal responses to punishment avoidance were observed in situations in which the value of the context was made explicit by instruction or overtraining^21^,^22^,^23^.

To test these hypotheses, healthy subjects underwent fMRI scanning while performing an instrumental learning task, involving multiple two-armed bandits (choice contexts) and followed by a post-learning assessment of option values. Two features of the task served our purposes: first, the task contrasted reward seeking with punishment avoidance learning; second, in specific choice contexts, we provided the information about the outcome of the foregone alternative—counterfactual information—to enhance relative value encoding^24^,^25^,^26^. We reasoned that presenting subjects with both the outcomes of the chosen and the unchosen options would facilitate the learning of the average value of the choice context (that is, the context value).

We found behavioural and neural evidence consistent with the idea that providing both the outcomes of the chosen and the unchosen options favoured the learning of a context-specific reference point. Behavioural results indicated that subjects learn similarly well reward seeking and punishment avoiding: a result that was efficiently captured by a computational model that embodies the idea of relative value learning. The same model was able to account for context dependence-induced valuation biases, as revealed by the post-learning test, specifically for options learnt in the presence of counterfactual feedback. fMRI analyses served two purposes. First, we used neural data to provide further experimental support to the computational analyses. Crucially model-based and model-free fMRI analyses concordantly indicated that neural activity in the brain valuation system was better explained assuming relative, than absolute value learning. Second, fMRI permitted us to reconcile previous discordant findings advocating for anatomical overlap or dissociation between reward seeking and punishment avoidance neural systems. In fact, the observed increase in contextual discrimination in the complete feedback conditions was followed by a shift in the neural encoding of negative outcomes from the insula to the ventral striatum.

## Results

### Experimental design

**Figure 1:**
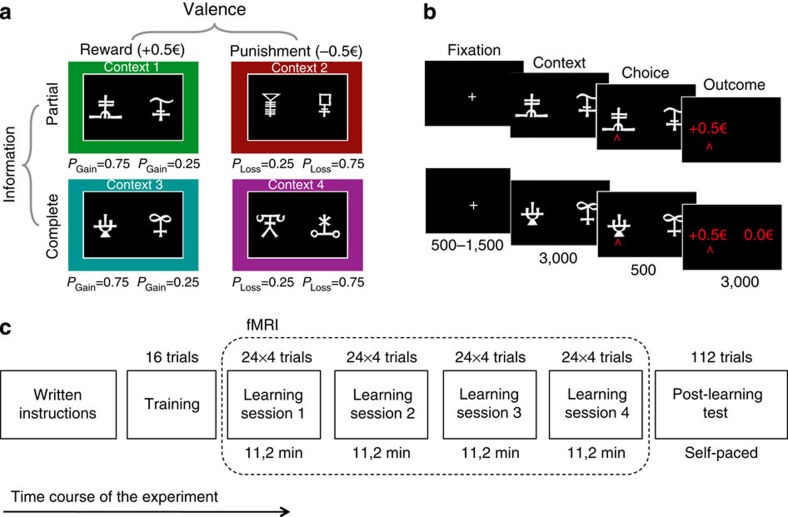
Experimental task and design. (**a**) Learning task 2 × 2 factorial design with 4 different contexts: reward/partial, punishment/partial, reward/complete, and punishment/complete. *P*_Gain_= probability of winning 0.5€; *P*_Loss_= probability of losing 0.5€. Note that the coloured frames are introduced in the figure for illustrative purposes, but were not present in the original task. (**b**) Successive screens of typical trials in the reward partial (top) and complete (bottom) contexts. Durations are given in milliseconds. (**c**) Time course of the experiment. Note that the post-learning test was uniquely based on the eight options of the last learning session.

Healthy subjects performed a probabilistic instrumental learning task with monetary gains and losses, adapted from those used in previous imaging, pharmacological and lesion studies^3^,^6^,^27^,^28^. The novel task presented a 2 × 2 factorial design with outcome valence (reward or punishment) and feedback information (partial or complete) as factors (Fig. 1a,b). In the learning task, options (materialized as abstract symbols) were always presented in fixed pairs. The fixed pairs of options represented stable choice contexts, with different overall expected value. In each context the two options were associated with different, but stationary, outcome probabilities, so that the subjects’ task was learning to choose the options associated either with highest reward probability or those associated with lowest punishment probability (correct options: *G*_75_ and *L*_25_ in the reward and the punishment context, respectively; incorrect options: *G*_25_ and *L*_75_ in the reward and the punishment context, respectively). Subjects performed four sessions of the task during fMRI scanning, each involving novel pairs of options. After the last session, subjects performed a post-learning test in which they were asked to indicate the option with the highest value, in choices involving all possible binary combinations—that is, including pairs of options that had never been associated during the task (Fig. 1c). As in previous studies, post-learning test choices were not followed by feedback, to not interfere with subjects’ final estimates of option values^29^,^30^.

### Instrumental performance

**Figure 2:**
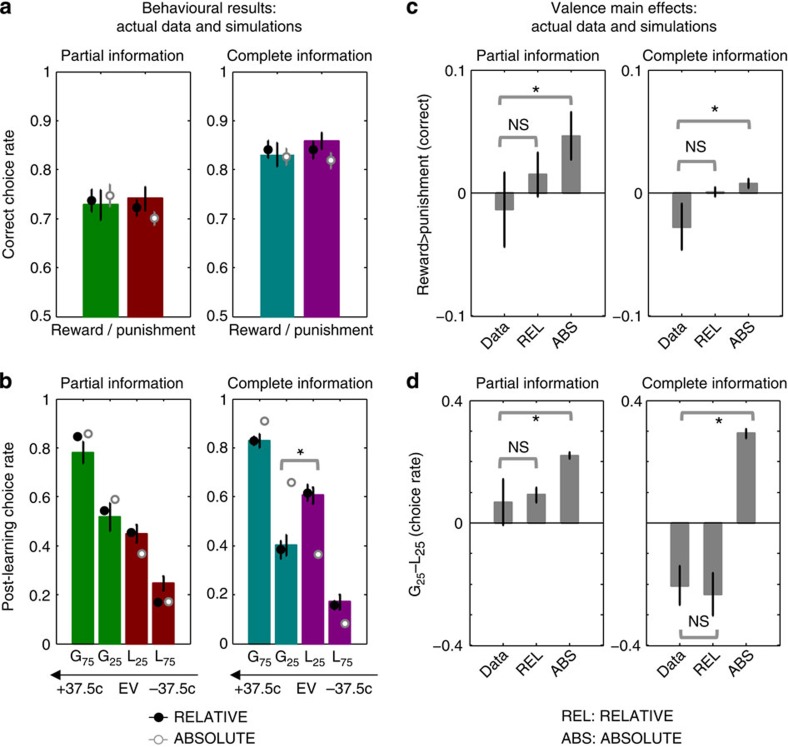
Behavioural results and model simulations. (**a**) Correct choice rate during the learning test. (**b**) Choice rate in the post-learning test. *G*_75_ and *G*_25_: options associated with 75% and 25% per cent of winning 0.5€, respectively; *L*_75_ and *L*_25_: options associated with 75% and 25% per cent of losing 0.5€, respectively. EV: absolute expected value (Probability(outcome) × Magnitude(outcome)) in a single trial. The values +37.5¢ and −37.5¢ correspond *G*_75_ and the *L*_75_ options, respectively. In **a** and **b** coloured bars represent the actual data and black (RELATIVE) and white (ABSOLUTE) dots represent the model simulated data. (**c**) Reward minus punishment correct choice rate during the learning test. (**d**) *G*_25_ minus *L*_25_ choice rate during the post learning test. *P<0.05 one sample *t*-test; NS, not significant (*N*=28). Error bars represent s.e.m.

We found significant evidence of instrumental learning (that is, participants sought rewards or avoided punishments; Table 1). Indeed, average correct response rate was significantly higher than chance level (that is, 0.5) in all contexts (*T*>7.0, *P*<0.001; Fig. 2a). A two-way analysis of variance (ANOVA) showed no effect of outcome valence (F=1.4, *P*>0.2), a significant effect of feedback information (F=30.7, *P*<0.001), and no significant interaction (F=0.7, *P*>0.7). Accordingly, *post hoc* investigation showed performances in complete feedback contexts as significantly higher compared with the partial feedback contexts (reward and punishment contexts: *T*>3, *P*<0.01). Thus, as in previous studies, healthy subjects learnt similarly from reward and punishments^3^,^29^, and efficiently integrated counterfactual information in instrumental learning^31^,^32^,^33^ (see Supplementary Note 1 and Supplementary Fig. 1 for reaction times data analysis).

### Post-learning choices

We found significant evidence of value retrieval during the post-learning test (Table 1)^29^,^30^. Indeed, a three-way ANOVA showed a significant effect of outcome valence (F=53.0, *P*<0.001) and a significant effect of option correctness (F=170.1, *P*<0.001), but no effect of feedback information (F=0.0, *P*>0.5; Fig. 2b). The only interaction that reached statistical significance was the correctness *x* feedback information (F=11.9, *P*<0.01). As for other interactions (double or triple), none reached statistical significance (all: F<2.0, *P*>0.1). A two-way ANOVA limited on the intermediate value options (that is, the less rewarding option in the reward contexts and the less-punishing option in the punishment contexts *G*_25_ and *L*_25_) with valence and feedback information as factors, crucially showed no significant effect of valence (F=1.6, *P*>0.2) nor of feedback information (F=0.2, *P*>0.2), but a significant interaction (F=9.4, *P*<0.01), thus reflecting an inversion in the evaluation of intermediate options, when moving from the partial to the complete feedback information contexts. More precisely, *post hoc* tests revealed that the percentage of choices towards the correct option of the punishment/complete context (*L*_25_) was higher compared with that towards the incorrect option in the reward/complete context (*G*_25_; T=3.2, *P*<0.01), despite their absolute expected value (EV; Probability(outcome) × Magnitude(outcome)) suggesting the opposite. (EV(*L*_25_)=−12.5¢; EV(*G*_25_)=+12.5¢). *Post hoc* analysis also showed a significantly different choice rates for the correct options in the reward compared with the punishment context (*G*_75_ versus *L*_25_ in both feedback information contexts: *T*>4.6, *P*<0.001), despite similar choice rate in the learning task (see also Supplementary Table 1). This indicated that post-learning choices could be explained neither by assuming that option values were encoded in an absolute manner, nor by assuming that they were merely reflecting past choice propensity (policy), but that they laid somehow halfway between these two extremes: a phenomenon that is parsimoniously explained by context-dependent option-value learning.

### Computational models

**Figure 3:**
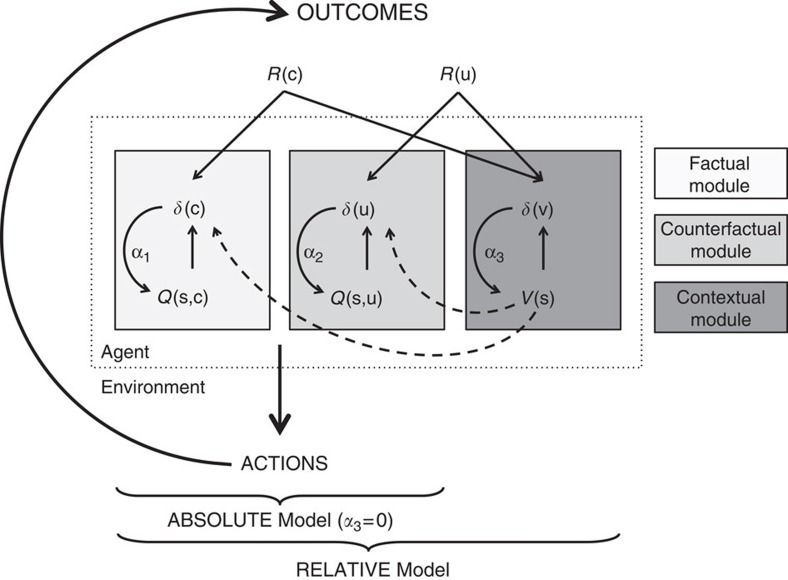
Computational architecture. The schematic illustrates the computational architecture used for data analysis. For each context (or state) ‘s’, the agent tracks option values (Q(s,:)), which are used to decide amongst alternative courses of action. In all contexts, the agent is informed about the outcome corresponding to the chosen option (R(c)), which is used to update the chosen option value (Q(s,c)) via a prediction error (*δ*(c)). This computational module (‘factual learning’) requires a learning rate (*α*_1_). In the complete feedback condition, the agent is also informed about the outcome of the unselected option (*R*(u)), which is used to update the unselected option value (*Q*(s,u)) via a prediction error (*δ*(u)). This computational module (‘counterfactual learning’) requires a specific learning rate (*α*_2_). In addition to tracking option value, the agent also tracks the value of the context (*V*(s)), which is also updated via a prediction error (*δ*(v)), integrating over all available feedback information (*R*(c) and *R*(u), in the complete feedback contexts and *Q*(s,u) in the partial feedback contexts). This computational module (‘contextual learning’) requires a specific learning rate (*α*_3_). The RELATIVE model can be reduced to the ABSOLUTE model by suppressing the contextual learning module (that is, assuming α_3_=0).

We fitted the behavioural data with model-free reinforcement-learning models (see Methods)^34^. The tested models included a standard Q-learning (thereafter referred to as ABSOLUTE), adapted to account for learning from counterfactual feedback, which has been most frequently used with this kind of task and we therefore consider as the reference model (hypothesis zero)^3^,^6^,^27^,^28^,^33^. We also considered a modified version of the ABSOLUTE model, which, similarly to other theories assumes that choice context (or state) values are separately learnt and represented^35^,^36^. The crucial feature of this model (thereafter referred to as RELATIVE) is that the context value sets the reference point to which an outcome should be compared before updating the option value; option values are therefore no longer encoded in an absolute, but in a relative scale (Fig. 3). The context value (*V*(s)) is defined as a ‘random-policy’ state value, aimed at capturing the overall expected value of a given pair of options, independent from subjects’ choice propensity. Note that the RELATIVE model shares a crucial feature (that is, relative option value encoding) with previous computational formulations, such as actor–critic and advantage learning models, that inspired its conception (see Supplementary Note 2 for additional model comparison including these preceding models and a discussion of their differences)^37^,^38^.

### Bayesian model selection

For each model, we estimated the free parameters by likelihood maximization (to calculate the Akaike Information Criterion, AIC, and the Bayesian Information Criterion, BIC) and by Laplace approximation of the model evidence (to calculate the exceedance probability; Tables 2 and 3). After *post hoc* analyses we found that the RELATIVE model better accounted for the data, both at fixed and random effect analysis (compared with the ABSOLUTE LL: *T*=4.1, *P*<0.001). This was also true when accounting (penalizing) for the different number of free parameters (AIC: *T*=3.4, *P*<0.001; BIC: *T*=2.1, *P*<0.05)^39^. We also calculated the exceedance probability (XP) of the model based on an approximate posterior probability of the model, and we consistently found that our model significantly outperformed the others (XP=1.0)^40^. Thus, context-dependent value encoding (RELATIVE) provided better account of learning test choices, even after correcting for its higher degrees of freedom (note that this conclusion was not affected by using different learning rates for the reward and the punishment contexts).

### Relative value encoding explains instrumental performance

**Figure 4:**
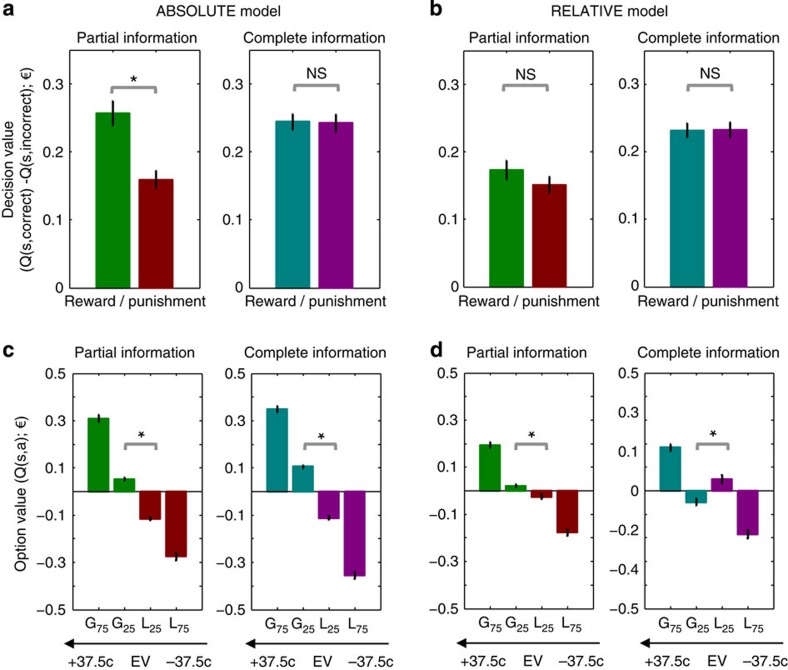
ABSOLUTE and RELATIVE model final value estimates. (**a**,**b**) The bars represent, for each model, the final optimal decision value estimates (the value of the correct minus the value of the incorrect option). (**c**,**d**): the bars represent, for each model, the final option value estimates.. *G*_75_ and *G*_25_: options associated with 75% and 25% per cent of winning 0.5€, respectively; *L*_75_ and *L*_25_: options associated with 75 and 25% per cent of losing 0.5€, respectively. EV: absolute expected value (Probability(outcome) * Magnitude(outcome)) in a single trial. The values +37.5¢ and −37.5¢ correspond *G*_75_ and the *L*_75_ options, respectively. The estimates are generated from individual history of choices and outcomes and subject-specific free parameters. *P<0.05 one sample *t*-test; ns: not significant (*N*=28). Error bars represent s.e.m.

To characterize the effect of context-dependent over absolute value learning, we generated for each trial *t* the probability of choosing the best option according to the models, given the subjects’ history of choices and outcomes at trial *t*−1 (Fig. 2a) and the individual best-fitting free parameters. We submitted model-simulated choice probabilities to the same statistical analyses reported above for their model-free counterpart. The RELATIVE model’s choices showed no effect of outcome valence (F=0.7, *P*>0.7), a significant effect of feedback information (F=53.4, *P*<0.001), and no significant interaction (F=0.7, *P*>0.4): the same statistical pattern as the actual data. The ABSOLUTE model choices displayed a significant effect of outcome valence (F=7.0, *P*<0.05), a significant effect of feedback information (F=43.1, *P*<0.001), and a significant interaction (F=4.2, *P*<0.05): a different statistical pattern compared to the actual data (Fig. 2c). A *post hoc* test showed lower performances in punishment/partial compared with the reward/partial context (*T*=2.4, *P*<0.05; Table 1). In fact, in the ABSOLUTE model, the model’s estimate of the decision value—defined as the difference between the correct and the incorrect option value—was significantly reduced in the punishment/partial compared with the reward/partial context (+15.9±1.2¢ versus +25.6±1.8¢; *T*=6.5, *P*<0.001; Fig. 4a). This naturally emerged from the reduced sampling of the *G*_25_ and *L*_75_ options respectively, induced by correct responding. This effect formally instantiates the computational problem inherent to punishment avoidance. This effect is not present in the RELATIVE model, in which, thanks to option value centring (that is, *R*_C,t_—*V*_t_(s) in *δ*_C_; and *R*_U,t_—*V*_t_(s) in *δ*_U_), decision values were similar in the reward and punishment domains (final decision values: +17.3±1.4¢ versus +15.1±1.2¢; *T*=1.7, *P*>0.1; Fig. 4b). Thus, as predicted from the analysis of model-derived option values, absolute value learning suffers from not being able to adequately fit symmetrical performances in the reward and punishment domains. The introduction of value contextualization proved sufficient to obviate this deficiency (Table 1 and Fig. 2c).

### Relative value encoding explains post-learning choices

To further probe the explanatory power of context-dependent (relative) over absolute value learning, we assessed and compared their ability to explain post-learning test choices (Fig. 2b). First, we found that the cumulative log-likelihood of the post-learning test was significantly higher assuming choices based on final option values obtained by the RELATIVE, compared with those by the ABSOLUTE model (−172.1±11.5 versus −220.3±16.7; *T*=7.0, *P*<0.001; predictive performances). Second, the post-learning choices simulated with the ABSOLUTE option values, produced a different behavioural pattern than the actual choices, specifically failing to capture the value inversion between intermediate value options (*G*_25_ and *L*_25_) in the complete feedback contexts (generative performances). Indeed, a two-way ANOVA on the RELATIVE simulated choices limited to the intermediate value options, with valence and feedback information as factors, showed, no significant main effect of valence (F=2.5, *P*>0.1), in line with actual data. The same analysis applied to ABSOLUTE simulated choices produced a significant effect of valence (F=660.2, *P*<0.001), contrary to actual data (Fig. 2d). *Post hoc* tests showed that the RELATIVE model fitted significantly higher choice rate for the complete *L*_25_ option compared with the complete *G*_25_ as observed in the behavioural data (*T*=3.4, *P*<0.001), whereas the ABSOLUTE model generated a significant opposite effect (*T*=19.2, *P*<0.001; Table 1). In fact, because of the additional (counterfactual) information provided to subjects, choice context values were better resolved in the complete compared with the partial feedback information contexts (final reward minus punishment context values: Δ*V*_Complete_=+33.6±2.3¢ versus Δ*V*_Partial_=+22.4±3.4¢; *T*=6.9, *P*<0.001; Supplementary Fig. 3A and 4A). As a direct consequence, contextual influences on option values were more pronounced in the complete feedback contexts. Indeed, intermediate value options (*G*_25_ and *L*_25_) in the complete feedback contexts displayed a more pronounced deviation from absolute expected value encoding (Fig. 4c,d). More precisely *G*_25_ options acquired a negative value (−4.8±1.6¢; *T*=2.9, *P*<0.01), whereas *L*_25_ a positive one (+4.9±1.7¢; *T*=3.0, *P*<0.01). Thus, as predicted from the analysis of model-derived option values, absolute value learning and encoding suffers from not being able to adequately fit the value inversion between intermediate value options in the complete context. Again, the introduction of value contextualization proved sufficient to obviate this deficiency (Table 1 and Fig. 2d).

### Neural Bayesian model selection

**Figure 5:**
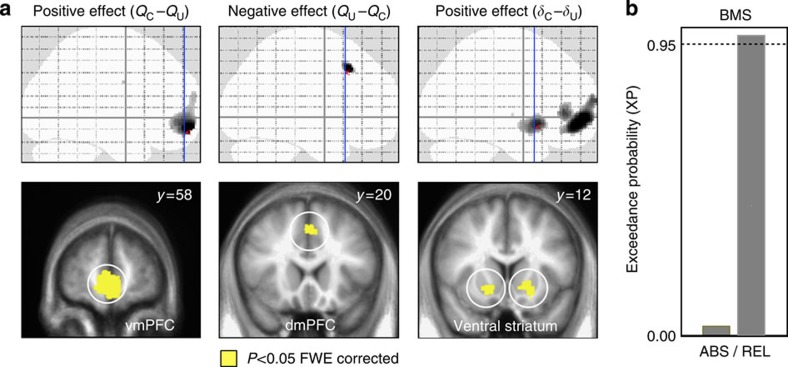
Neural model comparison. (**a**) Brain areas correlating positively and negatively with the difference between chosen and unchosen option value (*Q*_C_-*Q*_U_; left and central column), and correlating positively with the difference between chosen and unchosen prediction error (*δ*_C_–*δ*_U_; right column). Significant voxels are displayed on the glass brains (top) and superimposed to slices of the between-subjects averaged anatomical T1 (bottom). Coronal slices correspond to the blue lines on sagittal glass brains. Areas coloured in gray-to-black gradient on glass brains and in yellow on slices showed a significant effect (*P*<0.05, voxel level FWE corrected). *Y* coordinates are given in the MNI space. The results are from the GLM using the ABSOLUTE model parametric modulators (GLM1a). (**b**) Bayesian model comparison (BMS) of GLMs regressing ABSOLUTE (ABS) and RELATIVE (REL) option values and prediction errors (GLM1a and GLM1b). BMS is performed within the functional ROIs, presented on the left in yellow on the brain slices. Note that ROI selection avoids double dipping in favour of the hypothesis we aimed to validate, since the ROIs were defined from GLM1a (ABS) and GLM1a (ABS) was the hypothesis we aimed to reject.

After showing at multiple behavioural levels that option value contextualization occurs, we turned to corroborate this claim using model-based fMRI^41^. To achieve this, we devised a general linear model (GLM1) in which we modelled as separated events the choice onset and the outcome onset, each modulated by different parametric modulators: chosen and unchosen option values (*Q*_C_ and *Q*_U_) and prediction errors (*δ*_C_ and *δ*_U_). In a first GLM (GLM1a) we regressed the computational variables derived from the ABSOLUTE model. In a second GLM (GLM1b) we used the estimates from the RELATIVE model. We used the GLM1a to generate second level contrasts and, replicating previous findings, we found brain areas significantly correlating with the decision value (*Q*_C_–*Q*_U_) both positively (vmPFC) and negatively (dmPFC,), and brain areas correlating with the decision prediction error (*δ*_C_–*δ*_U_; vmPFC and ventral striatum: VS; *P*<0.05, whole brain family-wise error (FWE) corrected; Fig. 5a and Table 4; see also Supplementary Fig. 5)^3^,^27^,^42^,^43^. In a second step, we estimated within this prefrontal and striatal areas the same GLMs using Bayesian statistics. We found that the context-dependent value encoding (GLM1b) provided a significantly better account of the network’s neural activity (1,511 voxels; XP=0.97; Fig. 5b)^44^. Importantly this result also held true for each region of interest (ROI) separately (vmPFC: 936 voxels, XP=0.87; dmPFC: 71 voxels, XP=0.97; VS: 505 voxels, XP=0.93; for the RELATIVE model. Thus, replicating previous imaging findings implicating the medial prefrontal cortex and the striatum in value learning and decision making, we found that neural activity in these areas supports context-dependent (GLM1b) as opposed to absolute (GLM1a) value signals. Note that the ROIs were selected to favour the hypothesis that we want to reject (GLM1a)^45^.

### vmPFC activity is consistent with relative value encoding

**Figure 6:**
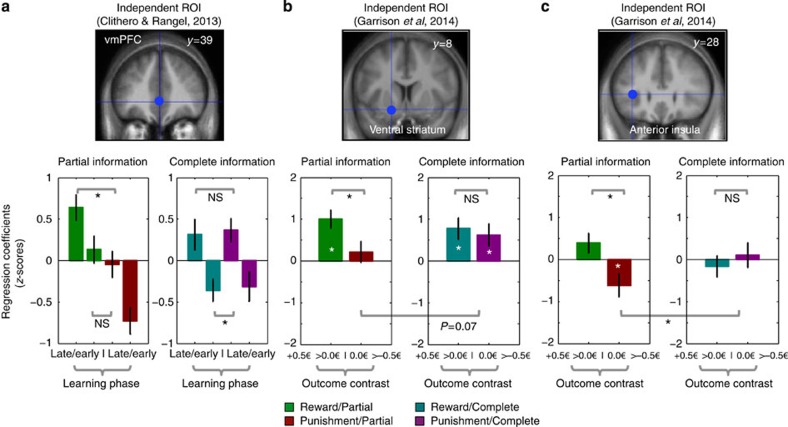
Model-free neural evidence of value contextualization. (**a**) Bars represent the regression coefficients extracted in the ventromedial prefrontal cortex, as a function of the task contexts (represented by different colours) and leaning phase (early: first eight trials; late: last eight trials). Regression coefficients are extracted from the model-free GLM2 within a sphere centered on literature-based coordinates of the ventromedial prefrontal cortex^11^. (**b**) & (**c**) Bars represent the regression coefficients for best>worst outcome contrast as a function of the task contexts. (‘+0.5€>0.0€’: best>worst outcome contrast in the reward contexts; ‘0.0€>−0.5€’: best>worst outcome contrast in the punishment contexts). Regression coefficients are extracted from the model-free GLM3 within spheres centered on literature-based coordinates of the striatum and anterior insula^8^. *Y* coordinates are given in the MNI space. Note that ROI selection avoids double dipping, since the ROIs were defined from independent studies (metanalyses). *P<0.05 one sample *t*-test comparing between regressors (black ‘*’) or to zero (white ‘*’; *N*=28); NS: not significant. Error bars represent s.e.m.

Model-based Bayesian fMRI analyses corroborated the RELATIVE model. To further support relative value encoding from the neural perspective, we also devised a categorical GLM (GLM2), in which choice events were modelled separately for each context and learning phase (early: first eight trials; late: last eight trials). For this analysis we focused on the vmPFC: the region that has been more robustly implicated in value encoding^11^,^12^. To avoid double dipping, we used a literature-based independent vmPFC ROI. On the basis of the model predictions and the assumption that the vmPFC represents values signals, we expected higher activation in the punishment/complete late trials (once the correct option *L*_25_ of the punishment/complete context has acquired a positive value), compared with reward/complete early trials (when the option values are not yet very different from zero). On the other side, we expected no such a difference in the partial contexts. To test this hypothesis we submitted the choice-related regression coefficients to a three-way ANOVA with valence (reward and punishment), feedback information (partial and complete) and learning phase (early and late) as factors (Fig. 6a). We found a significant main effect of phase (F=11.6, *P*<0.01), reflecting an overall learning-induced increase of vmPFC signal. We also found a significant main effect of valence (F=11.4, *P*<0.01), and a significant valence *x* information interaction (F=17.3, *P*<0.001), indicating that valence did not affect choice activations similarly in the partial and complete contexts, respectively. Consistent with this valence *x* information interaction, *post hoc* test indicated significant higher activations in the reward compared to the punishment late trials in the partial contexts (*T*=3.5, *P*<0.01), but no such difference in the complete contexts (*T*=0.7, *P*>0.4). Crucially and consistent with our predictions, *post hoc* test also indicated significant higher activations in the punishment early trials compared to the reward late trials in the complete contexts (*T*=3.8, *P*<0.001), but no such difference in the partial contexts (*T*=0.2, *P*>0.8). This result closely resembles that of option value inversion in the post-learning test. In summary, in addition to model-based fMRI analyses, we found that the activation pattern of the vmPFC is still consistent with relative, rather than absolute value encoding, also when analysed in a model-free manner.

### Outcome encoding is modulated by contextual discrimination

Previous fMRI and lesions studies, using similar behavioural tasks, suggest a role for the anterior insula (AI) in punishment learning, in contrast to that of the VS in the reward domain^3^,^6^,^20^,^46^,^47^,^48^. To challenge this hypothesis, we analysed outcome encoding within an anatomic mask including the insular cortex and the basal ganglia (Supplementary Fig. 6E). In the GLM (GLM3) used for this analyses, outcome events were modelled separately for each context and factual feedback value (*R*_C_). GLM3 was also ‘model-free’, since the categories were not derived from a computational model, but from the observable outcomes. We computed for each context separately a best>worst outcome contrast. Consistent with the neural opponency hypothesis and replicating previous findings, we found voxels in the VS significantly activated by the +0.5€>0.0€ contrast in the reward/partial context, thus encoding obtained rewards, and voxels in the AI significantly deactivated by the 0.0€>−0.5€ contrast in the punishment/partial context, thus encoding obtained punishments (*P*<0.05 FWE mask-level corrected; Supplementary Fig. 6A,B and Table 4). This functional dissociation still held at more permissive threshold of *P*<0.001 uncorrected, and after literature-based independent ROIs test^8^. In fact, to simultaneously and formally assess this functional dissociation as well as the effect of contextual information on outcome encoding, we submitted the outcome related contrasts to a three-way ANOVA with valence (reward and punishment) feedback information (partial and complete) and brain area (VS and AI) as factors (Fig. 6b,c). Indeed, ANOVA indicated a significant main effect of brain system (VS versus AI; F=45.8, *P*>0.001), which confirms the fact that outcomes are encoded with opposite signs in the two neural systems. We also found a significant main effect of feedback information (F=4.2, *P*<0.05) and a significant valence *x* information interaction (F=4.7, *P*<0.05), indicating that valence did not affect outcome signals similarly in the partial and complete contexts, respectively. *Post hoc* testing revealed significant differences in outcome encoding between the reward/partial and the punishment/partial contexts in both the AI (*T*=2.9, *P*<0.01), and the VS (*T*=2.3, *P*<0.05). Such differences were not observed when comparing the reward/complete to the punishment/complete contexts (*T*<0.7, *P*>0.4). Interestingly, *post hoc* tests also revealed that, in the complete feedback contexts, VS significantly encoded avoidance (*T*=2.4, *P*<0.05) and, concomitantly, the AI stopped responding to punishments (compared with the partial/punishment context: *T*=2.8, *P*<0.01). Finally, the triple valence *x* information *x* brain area interaction was not significant, reflecting the fact that the signal increases similarly in both areas when moving from the partial to the complete feedback contexts (in the striatum, from zero, it becomes positive; in the insula, from negative, it becomes zero; F=1.9; *P*>0.1).To further check that the result was not dependent on the (independent) ROI selection, we explored outcome related activations at an extremely permissive threshold (*P*<0.01 uncorrected), confirming no detectable implication of the AI in the punishment/complete context (Supplementary Fig. 6C,D). Altogether these results show that when additional information is provided (that is, complete feedback contexts), and therefore context value is better identified, punishment avoidance signals converge to the VS allowing the opponent system to ‘switch off’.

## Discussion

Healthy subjects performed an instrumental conditioning task, involving learning to maximize rewards and minimize punishments. Orthogonally to outcome valence, complete feedback information (the outcome of the chosen and the unchosen option) was provided to the subjects, in order to promote relative value encoding. The data displayed convergent evidence of option value contextualization at two independent behavioural levels: instrumental choices, and post-learning choices. First, punishment avoidance performances were matched to reward seeking ones, a result that cannot be explained by absolute value encoding; second, post-learning evaluation of the instrumental options, especially for those of the complete feedback contexts, displayed significant biases that can be parsimoniously explained assuming relative value encoding.

All these behavioural effects were submitted to computational model-based analyses. More specifically our analyses compared models representing two opposite views of the signals that drive decision-making: context-independent absolute value signals (that is, Q-learning) and context-dependent relative value signals (RELATIVE)^25^. We made a deliberate effort to keep these models as simple and parsimonious as possible. The RELATIVE model essentially tracks the mean of the distribution of values of the choice context (that is, the reference point) and uses it to centre option values. Notably, this model represents a minimal departure from a standard reinforcement learning algorithms that imply context or option values are updated with a delta rule, such as the Q-learning and actor–critic^34^. On the other side, the RELATIVE model can be seen as the most parsimonious algorithm implementation of a model that, departing from experienced raw values, learns to identify, for each situation (context) the ‘best’ and the ‘worst’ possible outcomes, based on an explicit representation of the underlying generative process (the task structure)^49^,^50^.

Punishment avoidance is computationally challenging. Simply stated: how can the instrumental response (avoid a punishment) be maintained despite the absence of further extrinsic reinforcement (punishment)? As already known and replicated here, absolute value learning methods are structurally not capable to cope with this problem^37^,^38^. In fact, the ABSOLUTE model predicted significant higher performances in the reward compared with the punishment context. Psychological models, such as the two-factor theory, suggested that a successful punishment avoidance could acquire a positive value and therefore act as intrinsic reinforcement to sustain learning^17^,^18^,^19^,^20^,^22^. The RELATIVE model embodies this idea by considering outcomes relative to the context in which they were delivered (*R*_C_–*V*). As a consequence of this feature, successful punishment avoidance (the neutral outcome 0.0€), acquired a positive value in the punishment avoidance context (where *V* is negative), providing a substrate for reinforcing the response. By doing so, it managed to equalize the performances between the reward and punishment context, as observed in human subjects.

We probed relative value encoding with an additional, and independent, behavioural measure. As in previous studies, we asked subjects to retrieve the value of the options after learning^29^,^30^. In this last task, options were presented in all possible combinations and were therefore extrapolated from their original choice context. Post-learning choices showed clear signs of value encoding. In fact, *G*_75_ choice rate was higher compared to *L*_25_ choice rate, despite the fact that their instrumental choice rate was similar. However, more in-depth analyses indicated that the behavioural pattern was more consistent with relative, rather than absolute value encoding. Subjects indeed failed to correctly retrieve the value of intermediate value options, to the point of preferring a lower value option (*L*_25_) to a higher value option (*G*_25_) in the complete feedback information, where relative value encoding was enhanced. Importantly, only the RELATIVE model was able to precisely capture this choice pattern (out-of-sample validation). The across task stability of relative value further corroborated our assumptions regarding the model, namely that value contextualization occurs within the learning rule and not within the policy. This effect is reminiscent of choice irrationality induced by context dependency (that is, preference reversal or ‘less is more’ effect) as if the adaptive function of value contextualization (in our case coping with punishment avoidance in the learning tasks) was traded against a bias of value estimates in the post-learning test^51^,^52^. Thus, as far as we were able to infer option values from choice data, they showed signs of context-dependency.

Replicating previous results, we found neural correlates of option values and prediction errors in a well-established reinforcement learning and decision-making network, including cortical and subcortical brain regions^3^,^27^,^42^,^43^. Relative and absolute value regressors shared a significant part of their variance due to the fact that both depend on the same history of choices and outcomes, and that the two models are structurally similar (nested) and similarly affected by task factors. Given these premises, to overcome this issue and corroborate relative value encoding, we implemented, as in recent studies, a neural model comparison analysis^53^,^54^. Bayesian model comparison showed that, within this prefrontal-striatal network, the context-dependent value-learning model (RELATIVE) provided a better explanation for BOLD responses than the ABSOLUTE model, which was used to generate the ROIs. Everything else being constant (individual history of choices and outcomes), the difference in model evidence could only be attributed to the value contextualization process itself, and therefore corroborates behavioural model selection. This model-based fMRI result has been backed up by a model-free analysis showing that signal changes in vmPFC (the core of the brain valuation system), once decomposed as a function of learning phase and task factors, displayed a pattern fully compatible with relative value encoding. More precisely we found that late vmPFC signal in punishment/complete context, was higher compared to the early signal reward/partial context: an effect that closely resembles that of the post-leaning value inversion.

Our finding of a functional double dissociation between the striatum and the insula in positive and negative outcome encoding perfectly replicates our previous results, and adds to a now critical mass of studies suggesting the existence of an opponent insular system dedicated to punishment-based learning and decision making^3^,^6^,^20^,^46^,^47^,^48^,^55^. Indeed, we found that the AI represented received negative outcomes in the punishment/partial context, in opposition to the pattern of activity in the ventral striatum, which represented received positive outcomes in the reward/partial condition. Strikingly, we found that in the punishment/complete context, negative outcome encoding in the AI faded, while the ventral striatum was concomitantly taking over. Globally, these results suggest that, by default, positive and negative values are represented in opposite directions by two opponent channels to ensure optimal outcome encoding in face of the impossibility of negative firing rates^56^,^57^,^58^. They also indicate that when relative valuation is facilitated (here in presence of complete feedback information), the ventral system is tuned to respond to ‘successful avoidance’ (intrinsic reinforcement) as it does for rewards^20^,^22^. This suggests that value contextualization can limit the need for simultaneously mobilizing multiple neural systems and therefore promotes neural parsimony. In our design, this effect was achieved in presence of the complete feedback information. Accordingly counterfactual outcome processing has been tightly associated to context-dependent (that is, relative) decision-making models, such as the regret theory^24^,^25^,^26^. However, it is nonetheless possible that in previous studies other task features, such as blocked design or explicit information about the outcome contingencies, could have concurred to reframe punishment avoidance tasks in order to induce the striatum to respond to successful avoidance^36^,^59^,^60^,^61^,^62^.

To summarize, our data suggest that as soon as an agent is engaged in a utility maximization-learning task, (s)he learns concomitantly the value of the available options and the value of the choice context in which they are encountered (the reference point). These quantities, option and context values, do not remain segregated but are rather integrated, so that the option value, originally encoded in an absolute scale, becomes relative to their choice context. Our study shows how value contextualization has the adaptive function of permitting efficient avoidance learning. Nevertheless, option value, being learned in a context-dependent manner, can produce suboptimal preferences (value inversion: irrational behaviour) when the options are extrapolated from their original choice context (for example, post learning). In the brain, value updating, supposedly achieved via prediction errors, is originally implemented by two different systems for the reward (reward system: ventral striatum) and the punishment (opponent system: anterior insula) domains, respectively, to obviate the difficulty to efficiently encode a large range of negative values. As a result of value contextualization, the reward responds to successful avoidance (*per se* a neutral outcome) and concomitantly the activity in the opponent system is suppressed.

## Methods

### Subjects

We tested 28 subjects (16 females; age 25.6±5.4 years). Power calculation studies suggested that a statistically valid sample size for fMRI study should be comprised of between 16 and 24 subjects^63^. We included *N*=28 subjects based on a pessimistic drop-out rate of 15%. We experienced no technical problems, so we were able to include all 28 subjects. Subjects were screened for the absence of any history of neurological and psychiatric disease or any current psychiatric medication, for right handedness and for normal or correct to normal vision. The research was carried out following the principles and guidelines for experiments including human participants provided in the declaration of Helsinki (1964). The local Ethical Committee of the University of Trento approved the study and subjects provided written informed consent prior to their inclusion. To sustain motivation throughout the experiment, subjects were remunerated according to the exact amount of money won in the experiment plus a fixed amount for their travel to the MRI center.

### Behavioural tasks

Subjects performed a probabilistic instrumental learning task adapted from previous imaging and patient studies^3^,^6^,^27^,^28^. Subjects were first provided with written instructions, which were reformulated orally if necessary (see Supplementary Note 3). They were informed that the aim of the task was to maximize their payoff, that reward seeking and punishment avoidance were equally important and that only factual (and not counterfactual) outcomes counted. Prior to entering the scanner, subjects performed a shorter (training) session, aimed to familiarize them with the task’s timing and responses. In the scanner subjects performed four learning sessions. Options were abstract symbols taken from the Agathodaimon alphabet. Each session contained eight novel options divided into four novel fixed pairs of options. The pairs of options were fixed so that a given option was always presented with the same other option. Thus, within each session, pairs of options represented stable choice contexts. Within sessions, each pair of options was presented 24 times for a total of 96 trials. The four option pairs corresponded to the four contexts (reward/partial, reward/complete, punishment/partial and punishment/complete), which were associated with different pairs of outcomes (reward contexts: winning 0.5€ versus nothing; punishment contexts: losing 0.5€ versus nothing) and a different quantity of information being given at feedback (partial and complete). In the partial feedback contexts, only the outcome about the chosen option was provided, while in the complete feedback contexts both the outcome of the chosen and the unchosen option were provided. Within each pair, the two options were associated to the two possible outcomes with reciprocal probabilities (0.75/0.25 and 0.25/0.75). During each trial, one option was randomly presented on the left and one on the right side of a central fixation cross. Pairs of options were presented in a pseudorandomized and unpredictable manner to the subject (intermixed design). The side on which a given option was presented was also pseudorandomized, such that a given option was presented an equal number of times in the left and the right of the central cross. Subjects were required to select between the two options by pressing one of the corresponding two buttons with their left or right thumb to select the leftmost or the rightmost option, respectively, within a 3,000 ms time window. After the choice window, a red pointer appeared below the selected option for 500 ms. At the end of the trial the options disappeared and the selected one was replaced by the outcome (‘+0.5€’, ‘0.0€’ or ‘−0.5€’) for 3,000 ms. In the complete information contexts, the outcome corresponding to the unchosen option (counterfactual) was also displayed. Note that between cues the outcome probability was truly independent on a trial-by-trial basis, even if it was anti-correlated in average. Thus, in a complete feedback trial, subjects could observe the same outcome from both cues on 37.5% of trials and different outcomes from each cue on 62.5% of trials. A novel trial started after a fixation screen (1,000 ms, jittered between 500–1,500 ms). During the anatomical scan and after the four sessions subjects performed a post-learning assessment of option value. This task involved only the 8 options (2 × 4 pairs) of the last session, which were presented in all possible pair-wise combinations (28, not including pairs formed by the same option)^29^,^30^. Each pair of options was presented 4 times, leading to a total of 112 trials. Instructions were provided orally after the end of the last learning session. Subjects were informed that they would be presented pairs of options taken from the last session, and that all pairs had not necessarily been displayed together before. During each trial, they had to indicate the option with the highest value by pressing on the buttons as they had done during the learning task. Subjects were also advised that there was no money at stake, but encouraged to respond as they would have if that were the case. In order to prevent explicit memorizing strategies, subjects were not informed that they would have performed this task until the end of the fourth (last) session of the learning test. Timing of the post-test differed from the learning test in that the choice was self-paced and in the absence of the outcome phase.

### Behavioural analyses

From the learning test, we extracted the choice rate as dependent variable. Statistical analyses were performed on the percentage of correct choices, i.e., choices directed toward the most advantageous stimulus (most rewarding or the less punishing), sorted as a function of the context (see Behavioral tasks). Statistical effects were assessed using two-way repeated-measures ANOVA with (1) feedback information and (2) feedback valence as factors. Between-context differences in correct responses were also tested *post hoc* using a two-sided, one-sample *t*-test. Reaction times were also extracted from the learning test and submitted to the same factorial analyses used for the correct choice rate (see Supplementary Note 1 and Supplementary Fig. 1). Choice rate was also extracted from the post-learning test and sorted for each option separately, as the percentage of choice toward a given stimulus taking into account all possible comparisons. Post-learning choice rate was submitted to three-way repeated-measures ANOVA, to assess the effects of (1) feedback information, (2) feedback valence and (3) option correctness. We also performed a two-way repeated-measures ANOVA focused on the intermediate value options, assessing the effect of (1) feedback information and (2) valence. Between-option differences in post-learning choices were tested *post hoc* using a two-sided, one-sample *t*-test. As a control analysis, the percentage of direct choices involving the *G*_25_ and the *L*_25_ cues (that is, the intermediate value cues) has also been analysed separately for each comparison (see Supplementary Note 1 and Supplementary Fig. 2). All statistical analyses were performed using Matlab (www.mathworks.com) with the addition of the Statistical toolbox and other free-download functions (rm_anova2.m, RMAOV33.m).

### Computational models

We analysed our data with model-free reinforcement learning algorithms^34^. The goal of all models was to find in each choice context (state: s) the option that maximizes the cumulative reward R. We compared two alternative computational models: a Q-learning model, extended to account for counterfactual learning (ABSOLUTE), which instantiates ‘absolute value-based’ learning and decision making by learning option values independently of the choice context in which they are presented^25^,^34^; the RELATIVE model which learns option values relative to the choice context in which they are presented^35^,^36^,^37^,^38^,^64^ (Fig. 3).

(1) ABSOLUTE model

At trial t the chosen (c) option value of the current context (s) is updated with the Rescorla-Wagner rule (also called delta-rule)^65^:




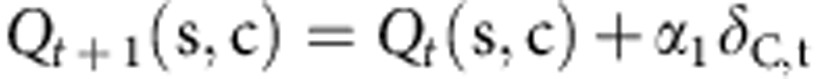




and




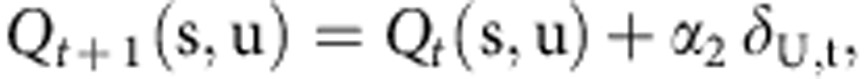




where *α*_1_ is the learning rate for the chosen option and *α*_2_ the learning rate for the unchosen (u) option (counterfactual learning rate). *δ*_C_ and *δ*_U_ are prediction error terms calculated as follows:




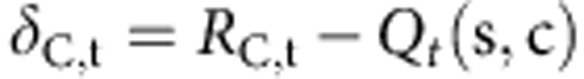




(update in both the partial and complete feedback contexts) and




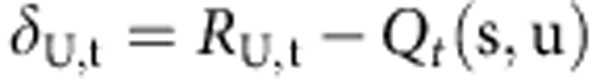




(in the complete feedback contexts only).

(2) RELATIVE model

We also devised a new model (RELATIVE), which, instantiates the ‘relative value-based’ learning and decision-making. The key idea behind RELATIVE model is that it separately learns and tracks the choice context value (*V*(s)), used as the reference point to which an outcome should be compared before updating option values. Previous algorithms, such the actor-critic and the advantage learning model, inspired the RELATIVE model (see Supplementary Note 2, Supplementary Fig. 3 and Supplementary Table 3 for additional model comparison analyses including the actor-critic model). All these models implement relative value learning of option values, based on *V*(s) estimates. The RELATIVE model differs in that it is extended to account for counterfactual feedback and that V(s) is learnt in an ‘random-policy’ manner (that is, the state value is independent from the policy followed by the subject. (see Supplementary Note 2, Supplementary Fig. 4 and Supplementary Table 4 for additional model comparison analyses supporting these assumptions). Crucially *V*(s) is not merely the choice-probability weighted sum of options’ value, but rather affects (controls) them. In fact *V*(s) is used to centre option prediction errors as follows:




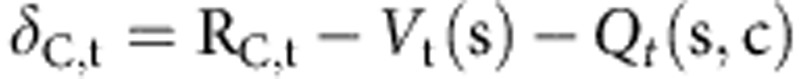




and




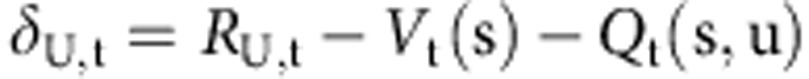




(in the complete feedback contexts only). As a consequence the option values are no longer calculated in an absolute scale, but relatively to their choice context value *V*(s). Context value is also learned via a delta rule:




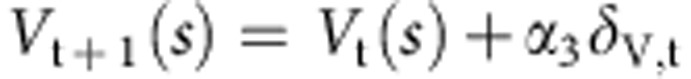




Where *α*_*3*_ is the context value learning rate and *δ*_V_ is a prediction error-term calculated as follows:




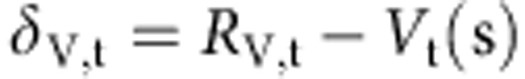




where *t* is the number of trials and *R*_V_ is the context-level outcome at trial *t*: a global measure that encompasses both the chosen and unchosen options. In the complete feedback contexts the average outcome trial (*R*_V_) is calculated as the average of the factual and the counterfactual outcomes as follows:




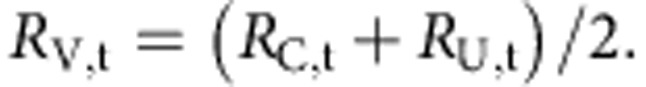




Given that the average outcome trial (*R*_V_) is meant to be a context-level measure, in order to incorporate unchosen option value information in *R*_V_ also in the partial feedback contexts, we considered *Q*_t_(s,u) a good proxy of *R*_U,t_ and calculated *R*_V,t_ as follows (see Supplementary Note 2 and Supplementary Table 2 for model comparison justifications of these assumptions):




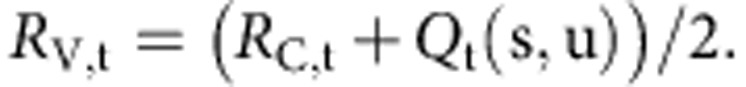




To sum up, our model space included 2 models: the ABSOLUTE model (Q-learning) and the RELATIVE model. In all models decision-making relied on a softmax function:




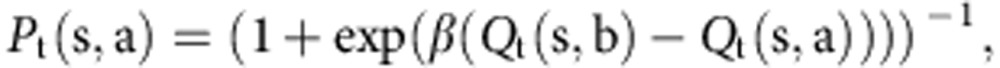




where *β* is the inverse temperature parameter. The Matlab codes implementing the computational models are available upon request to the corresponding author.

### Parameters optimization and model selection procedures

We optimized model parameters, the temperature (*β*), the factual (*α*_1_), the counterfactual (*α*_2_) and the contextual (*α*_3_) learning rates (in the RALATIVE model only), by minimizing the negative log likelihood (LL_max_) and (in a separate optimization procedure) the negative log of posterior probability (LPP) of the data given different parameters settings using Matlab’s fmincon function, initialized at multiple starting points of the parameter space, as previously described^66^,^67^. Negative log-likelihoods (LL_max_) were used to compute classical model selection criteria. The LPP was used to compute the exceedance probability and the expected frequencies of the model.

We computed at the individual level (random effects) the Akaike’s information criterion (AIC),




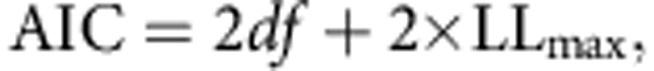




the Bayesian information criterion (BIC),




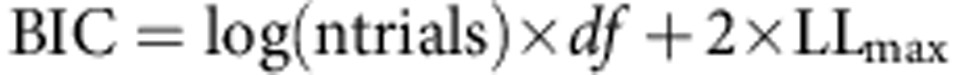




and the Laplace approximation to the model evidence (LPP);




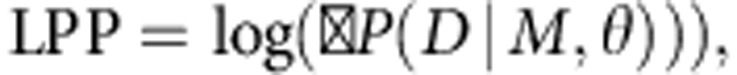




where *D, M* and *θ* represent the data, model and model parameters respectively. *P*(*θ*_n_) is calculated based on the parameters value retrieved from the parameter optimization procedure, assuming learning rates beta distributed (betapdf(parameter,1.1,1.1)) and softmax temperature gamma-distributed (gampdf(parameter,1.2,5))^68^. The present distributions have been chosen to be relatively flat over the range of parameters retrieved in the previous and present studies. The LPP increases with the likelihood (which measures the accuracy of the fit) and is penalized by the integration over the parameter space (which measures the complexity of the model). The LPP, as the BIC or AIC, thus represent a trade-off between accuracy and complexity and can guide model selection. Individual LPPs were fed to the mbb-vb-toolbox (https://code.google.com/p/mbb-vb-toolbox/)^40^. This procedure estimates the expected frequencies of the model (denoted PP) and the exceedance probability (denoted XP) for each model within a set of models, given the data gathered from all subjects. Expected frequency quantifies the posterior probability, i.e., the probability that the model generated the data for any randomly selected subject. This quantity must be compared to chance level (one over the number of models in the search space). Exceedance probability quantifies the belief that the model is more likely than all the other models of the set, or in other words, the confidence in the model having the highest expected frequency. We considered the ‘best model’, the model which positively fulfilled all the criteria.

### Model simulation analyses

Once we had optimized models’ parameters, we analysed their generative performance by analysing the model simulation of the data^69^. Model estimates of choice probability were generated trial-by-trial using the best individual parameters in the individual history of choices and outcomes. Model choice probability was then submitted to the same statistical analysis as the actual choices. The evaluation of generative performances involved two steps: first, the assessment of the model’s ability to reproduce the key statistical effects of the data; second, the assessment of the model’s ability to match subjects’ choices. The first step essentially involved within-simulated data comparisons, in both the form of ANOVA and *post hoc* one-sample *t*-test. The second step involved comparison between simulated and actual data with a one-sample *t*-test, and adjusting the significance level for the multiple comparisons (see the results reported in Table 1). We also tested models’ performances out of the sample by assessing their ability to account for post-learning test choices. Concerning the post-learning test analysis, under the assumption that choices in the post-learning test were dependent on the final option values, we calculated the probability of choice in the post-learning test using a softmax, using the same individual choice temperature optimized during the learning test (note that similar results have been obtained when optimizing a β specific to the post-learning test). On the basis of model-estimate choice probability, we calculated the log-likelihood of post-learning choices that we compared between computational models. Finally, we submitted the model-estimate post-learning choice probability to the same statistical analyses as the actual choices (ANOVA and *post hoc t*-test; within-simulated data comparison) and we compared modelled choices to the actual data (pair-wise comparisons, corrected for multiple comparisons; Table 1).

### fMRI data acquisition and preprocessing

A 4T Bruker MedSpec Biospin MR scanner (CiMEC, Trento, Italy) and an eight-channel head coil were used to acquire both high resolution T1-weighted anatomical MRI using a 3D MPRAGE with a resolution of 1 mm^3^ voxel and T2*-weighted Echo planar imaging (EPI). The parameters of the acquisition were the following, 47 slices acquired in ascending interleaved order, the in-plan resolution was 3 mm^3^ voxels, the repetition time 2.2 s, and the echo time was 21 ms. A tilted plane acquisition sequence was used to optimize functional sensitivity to the orbitofrontal cortex^70^. The acquisition started from the inferior surface of the temporal lobe. This implicated that, in most subjects, the acquired volume did not include the inferior part of the cerebellum. Preprocessing of the T1-weighted structural images consisted in coregistration with the mean EPI, segmentation and normalization to a standard T1 template, and average across all subjects to allow group-level anatomical localization. Preprocessing of EPI consisted in spatial realignment, normalization using the same transformation as structural images, and spatial smoothing using a Gaussian kernel with a full width a half-maximum of 8 mm. Final voxel size was 2 mm^3^. Preprocessing was realized using SPM8 (www.fil.ion.ucl.ac.uk).

### fMRI data analyses

EPI images were analysed in an event-related manner within the general linear model (GLM) framework, using SPM8 software. In GLM1, each trial was modelled as having two time points, corresponding to choice and outcome display onsets, modelled by two separate regressors. Choice onset and outcome onset were then modulated with different parametric regressors. In order to account for irrelevant motor or visual activations, the first parametric modulators for GLM1 were: (1) the response (coded as 1 and −1, for the right or left response, respectively) for the choice onset, and (2) the number of outcomes on the screen (codes as 1 and 2 for the partial and complete feedback context, respectively) for the outcome onset. These control parametric modulators generated motor and visual activations (data not shown). To correct for motion artifact, all GLMs also included the subject/session specific realignment parameters as nuisance covariates. The GLM1a and GLM1b differed in the computational model used to generate the parametric modulators. In addition to motor and visual regressors, in GLM1 the choice onsets were modulated by the trial-by-trial estimates of *Q*_C_ and *Q*_U_, whereas the outcome onsets by the trial-by-trial estimates of *δ*_C_ and *δ*_U_. In the partial feedback trials, the unchosen prediction error regressor (*δ*_U_) was systematically set at zero. Computational regressors were generated for each subject using the group level mean of the best individual parameters and the individual history of choices and outcomes. Regressors were z-scored before regression in order to ensure between-model, between-subject and between-modulator commensurability of the regression coefficients (Table 3). The computational variables of the GLM1a were derived from the ABSOLUTE computational model. GLM1b was structurally identical to GLM1a, except for the fact that the computational variables were derived from the RELATIVE model. All activations concerning GLM1 reported in the figure 5 survived a threshold of *P*<0.05 with voxel level whole brain FWE correction for multiple comparisons. In GLM2, each trial was modelled as having one-time points, corresponding to the stimulus display onsets. The choice onsets were split into eight different events (categories) as a function of task factors (feedback information x outcome valence) and the position of the trial within the learning curve (early: first eight trials; late: last eight trials; we did not include the mid eight trials so as to only include in each category trials belonging as clearly as possible to the ‘incremental’ versus the ‘plateau’ phase of the learning curves). In GLM3, each trial was modelled as having one-time points, corresponding to the outcome display onsets. The outcome onsets were split into eight different events (categories) as a function of the task factors (feedback information x outcome valence) and obtained outcome (*R*_C_). We computed at the first level a best>worst outcome contrast for each context separately (‘+0.5€>0.0€’: best>worst outcome contrast in the reward contexts; ‘0.0€>−0.5€’: best>worst outcome contrast in the punishment contexts). All GLMs were estimated using classical statistical techniques and linear contrast of parametric modulators were computed at the individual level and then taken to a group-level random effect analysis (one-sample *t*-test). Based on our hypotheses, second level contrasts of GLM3 were estimated within an anatomic mask encompassing bilaterally the insula and the basal ganglia (caudate, putamen and pallidum; >9 × 10^5^ voxels of 2 mm^3^) (Supplementary Fig. 6E). The mask has been designed using MARINA software (http://www.fil.ion.ucl.ac.uk/spm/ext/). Activations concerning GLM3 and reported in yellow in Supplementary Fig. 6 survived a threshold of *P*<0.05 with voxel level anatomic mask FWE correction, that is, the multiple comparison accounted for the number of voxels in the mask rather than the whole brain (small volume correction). Activations are reported in the coordinates space of the Montreal Neurology Institute (MNI). Activations were anatomically labelled using the Brodmann and the automatic anatomical labelling template implemented by the software MRIcron (www.mccauslandcenter.sc.edu/mricro).

### Region of interest analyses

ROI analyses served three purposes: (1) assess and compare the goodness of fit of the neural data between the RELATIVE and the ABSOLUTE computational model parametric modulators (GLM1); (2) assess choice related brain activity in the vmPFC as a function of the task contexts (GLM2); (3) assess outcome encoding in the VS and the AI as a function of the task contexts (GLM3). All ROI analyses were designed to avoid double dipping in favour of the hypothesis we aimed to validate^45^. To assess goodness of fit (neural model selection), we first defined from GLM1a (ABSOLUTE’s regressors) a ‘task network’ mask including all the voxels which survived cluster level *P*<0.05 (FWE corrected) in the following contrasts: positive and negative correlation with ‘*Q*_C_–*Q*_U_’ (decision value) and ‘*δ*_C_—*δ*_U_’ (decision prediction error) (see Fig. 5a). Within this mask (total voxels number=1,511), we estimated GLM1a and GLM1b (best model regressors) using Bayesian statistics, which provided log evidence for each GLM. Log evidence was then fed to BMS random effects analysis, which computed the exceedance probability of each GLM within the mask^44^. This analysis indicates which GLM better explained the neural data. To avoid double dipping in favour of the hypothesis that we wanted to support, we selected the ROIs, which favoured the hypothesis we wanted to reject (GLM1a, ABSOLUTE model)^45^. The second ROI analysis was devoted to study how task factors (contexts) affected choice related activity. A spherical ROI of 4 mm diameter was centred on ventromedial prefrontal coordinates reported to be significantly associated with decision value in a recent meta-analysis^11^. Regression coefficients from the GLM2 were submitted to a repeated measure three-way ANOVA analysis with valence (reward and punishment), feedback information (partial and complete) and learning phase (early, late) as factors. The third ROI analysis was devoted to study how task factors (contexts) affected outcome encoding. Spherical ROIs of 4 mm were centered on striatal (VS) and insular (AI) coordinates reported to be significantly associated with reward and punishment prediction errors in a recent meta-analysis^8^. Regression coefficients were submitted to a repeated measure three-way ANOVA analysis with neural system (VS or AI) and valence (reward and punishment) and feedback information (partial and complete) as factors. In the second and third ROI analyses the *post hoc* significance assessed with two-sided one-sample *t*-test.

## Additional information

**How to cite this article:** Palminteri, S. *et al.* Contextual modulation of value signals in reward and punishment learning. *Nat. Commun.* 6:8096 doi: 10.1038/ncomms9096 (2012).

## Supplementary Material

Supplementary Information Supplementary Figures 1-6, Supplementary Tables 1-4, and Supplementary Notes 1-3, and Supplementary References

## Figures and Tables

**Table 1:** Experimental and computational model-derived variables.

**Dependent variables**	**DATA**	**ABSOLUTE**	**RELATIVE**
Learning test: correct choice rate			
Reward partial (% correct)	0.73±0.03	0.75±0.02	0.74±0.02
Punishment partial (% correct)	0.74±0.03	0.70±0.02	0.72±0.02
Reward complete (% correct)	0.83±0.02	0.83±0.02	0.84±0.02
Punishment complete (% correct)	0.86±0.02	0.81±0.02*	0.84±0.02
			
Post-learning test: choice rate			
G_75_ partial (% choices)	0.78±0.04	0.86±0.01	0.85±0.01
G_25_ partial (% choices)	0.51±0.06	0.58±0.01	0.54±0.01
L_25_ partial (% choices)	0.45±0.04	0.37±0.01	0.45±0.01
L_75_ partial (% choices)	0.25±0.03	0.17±0.01	0.16±0.01
G_75_ complete (% choices)	0.83±0.03	0.91±0.01	0.83±0.02
G_25_ complete (% choices)	0.40±0.04	0.66±0.01*	0.38±0.03
L_25_ complete (% choices)	0.61±0.03	0.37±0.01*	0.62±0.03
L_75_ complete (% choices)	0.17±0.03	0.08±0.01	0.16±0.02

ABSOLUTE, absolute value learning model; DATA, experimental data; RELATIVE, relative value learning model (best-fitting model).

The table summarizes for both tasks their experimental and model-derived dependent variables. Data are expressed as mean±s.e.m.

^*^P<0.05, *t*-test, comparing the model-derived values with the actual data after correcting for multiple comparisons (*N*=28).

**Table 2:** Model comparison criteria.

**Model**	**DF**	−2*LLmax	2*AIC	**BIC**	−2*LPP	**PP**	**XP**
ABSOLUTE	3	307±20	319±20	325±20	314±20	0.08±0.03	0.0
RELATIVE	4	295±22	311±22	319±22	304±21	0.92±0.03	1.0

AIC, Akaike Information Criterion (computed with LLmax); BIC, Bayesian Information Criterion (computed with LLmax); DF, degrees of freedom; LLmax, maximal log likelihood; LPP, log of posterior probability; PP, posterior probability of the model given the data; XP, exceedance probability (computed from LPP).

The table summarizes for each model its fitting performances.

**Table 3:** Computational free parameters.

	**LL maximization**		**LPP maximization**	
**Free parameter**	**ABSOLUTE**	**RELATIVE**	**ABSOLUTE**	**RELATIVE**
Inverse temperature (*β*)	17.4±5.92	21.52±5.95	11.4±0.97*	13.66±1.32*
Factual learning rate (*α*_1_)	0.28±0.02	0.19±0.02	0.29±0.02*	0.20±0.01*
Counterfactual learning rate (*α*_2_)	0.18±0.02	0.15±0.02	0.20±0.02*	0.16±0.02*
Context learning rate (*α*_3_)	—	0.33±0.07	—	0.34±0.07*

ABSOLUTE, absolute value learning model; RELATIVE, relative value learning model (best-fitting model); LL maximization, parameters obtained when maximizing the negative log likelihood; LPP maximization, parameters obtained when maximizing the negative log of the Laplace approximation of the posterior probability.

The table summarizes for each model the likelihood maximizing (‘best’) parameters averaged across subjects. Data are expressed as mean±s.e.m.

The average values retrieved from the LL maximization procedure are those used to generate the parametric modulators of GLM1a and GLM1b.

^*^P<0.001 when correlating the LPP-based with LL-based free parameters (robust regression, *N*=28).

**Table 4:** Brain activations.

**Contrast**	**Label**	[***x y z*** ]	**BA**	**AAL**	***T***	***S***	**GLM**
*Q*_C_–*Q*_U_							
	vmPFC	[4 58 −12]	10,11	Medial frontal gyrus, pars orbitalis	6.57	939	1
*Q*_U_–*Q*_C_							
	dmPFC	[−6 20 42]	8,32	Superior medial frontal gyrus	5.63	71	1
*δ*_C_–*δ*_U_							
	vmPFC	[−6 54 −4]	10,11	Medial frontal gyrus (pars orbitalis)	7.00	1226	1
	vlPFC	[−52 36 2]	45,47	Inferior frontal gyrus (pars triangularis)	5.79	119	1
	Left-VS	[−16 12 −8]	—	Putamen, pallidum	4.78	271	1
	Right-VS	[14 10 −8]	—	Putamen, pallidum	4.57	234	1
+0.5€>0.0€ (reward/partial)							
	Right-VS	[16 12 −6]	—	Putamen, pallidum	3.89	21	3
−0.5€>0.0€ (punishment/partial)							
	AI	[16 12 −6]	48	Insula	4.02	43	3

AAL, automatic anatomic labelling; AI, anterior insula; BA, Brodmann area; dmPFC; dorso-medial prefrontal cortex; GLM, general linear model; *S*, size of the activation (voxels); *T*, *t*-values of the maxima; vmPFC, ventro-medial prefrontal cortex; VS, ventral striatum; [*x y z*], MNI coordinates.

The table summarizes brain activations reported in Fig 5a and Supplementary Fig. 6a,b, significant at *P*<0.05 FWE whole brain-level (GLM1a) or anatomic mask-level (GLM3) FWE corrected (one-sample *t*-test; *N*=28).
