# Supplementary material for: Contextual modulation of value signals in reward and punishment learning
Source: Nat Commun. 2015 Aug 25;6:8096. doi: 10.1038/ncomms9096 (PMC4560823; doi:10.1038/ncomms9096)
Supplement: Supplementary Information — Supplementary Figures 1-6, Supplementary Tables 1-4, and Supplementary Notes 1-3, and Supplementary References [file ncomms9096-s1.pdf]

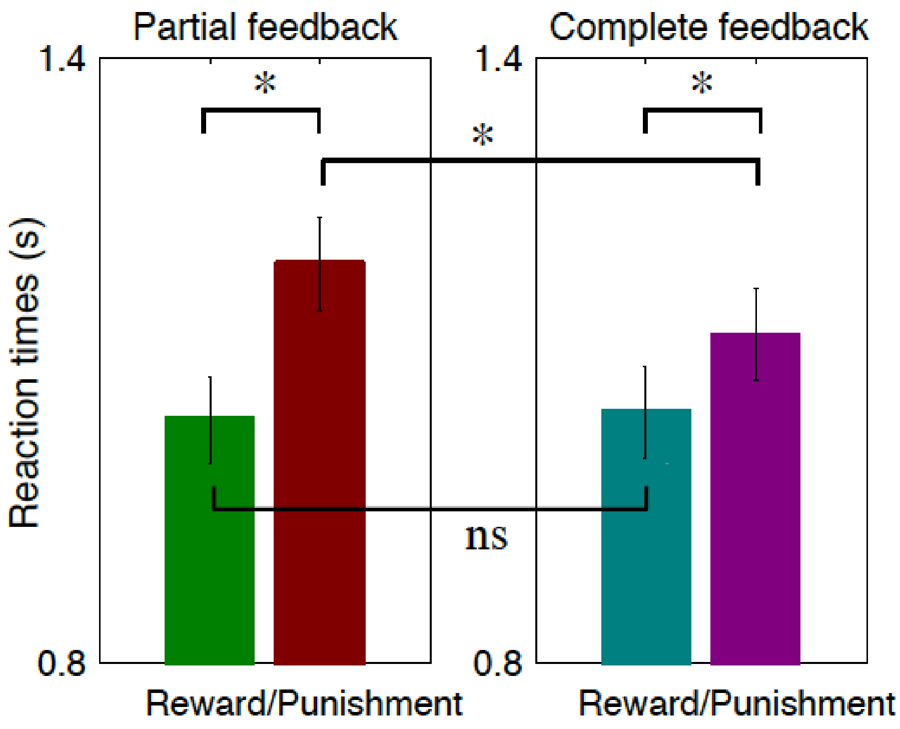


**Supplementary Figure 1: reaction times.**

Average reaction times during the learning test as a function of the choice contexts. *P<0.05 one sample t-test; ns: not significant. Error bars represent s.e.m.

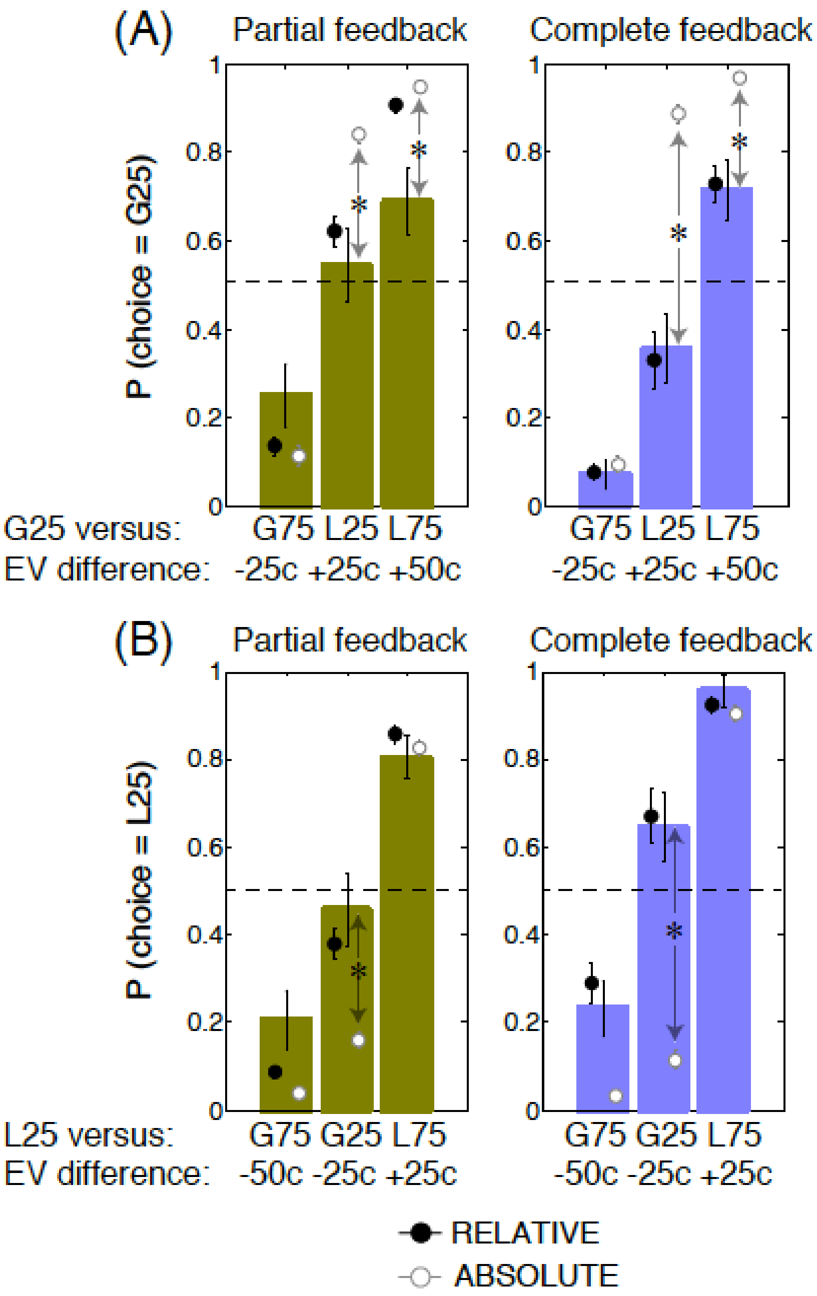


**Supplementary Figure 2: intermediate value cues post learning choice rates**

(A) and (B) Post-learning choice rate for comparisons involving the incorrect option in reward conditions ($G_{25}$: options associated with 25% percent of winning 0.5€) and the correct option in the punishment conditions ($L_{25}$: options associated with 25% percent of losing 0.5€), respectively. EV difference: difference in the absolute expected value (Probability(outcome) * Magnitude(outcome)) for a given cues comparison. Negative "EV difference" values indicate lower EV in the intermediate value cue ($G_{25}$ or $L_{25}$) compared to the cue to which it is compared. Positive "EV difference" values indicate the opposite. Colored bars represent the actual data and black (RELATIVE) and white (ABSOLUTE) dots represent the model-simulated data. *P<0.05 one sample t-test corrected for multiple (twelve) comparisons. Error bars represent s.e.m.

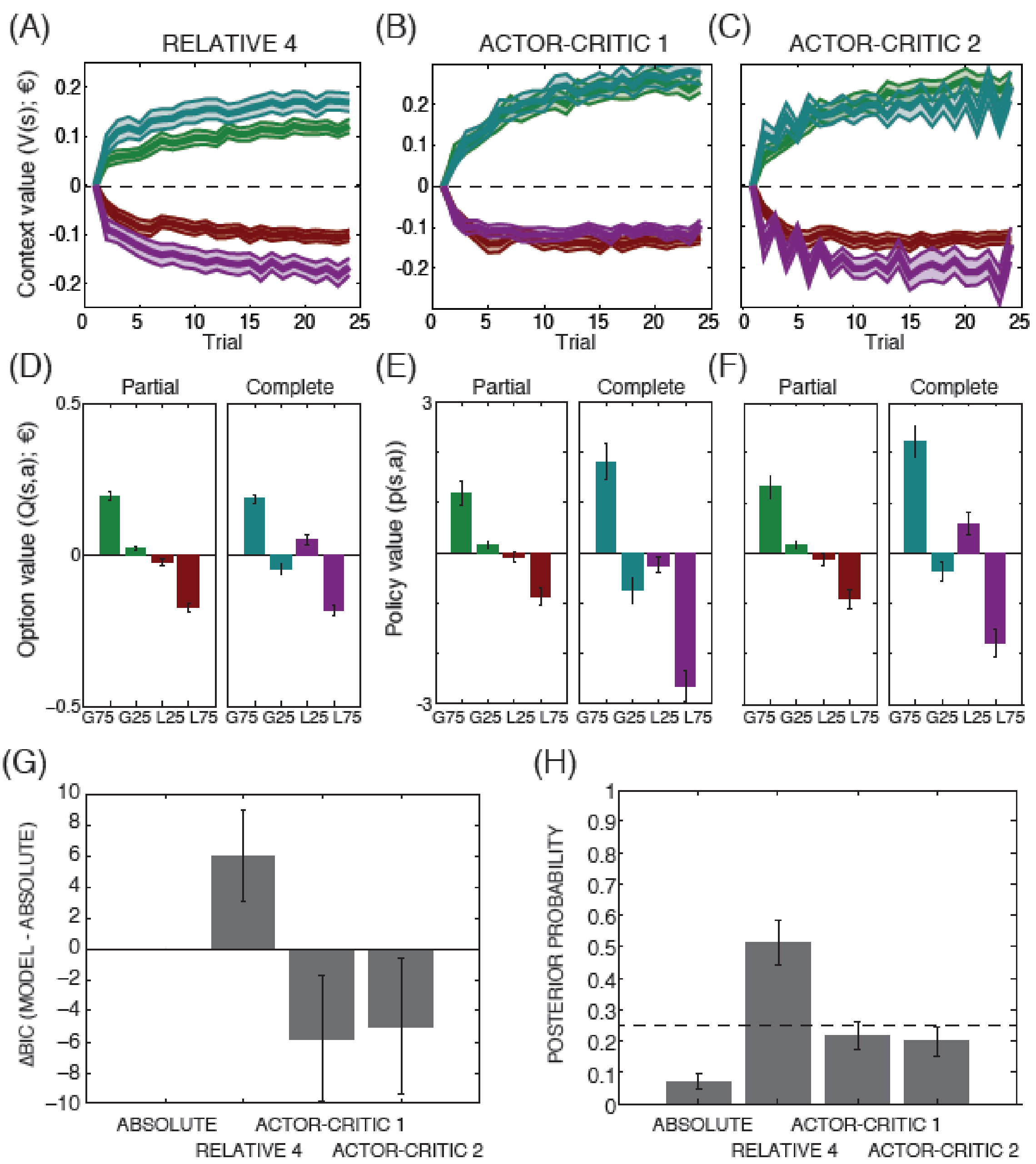


**Supplementary Figure 3: comparison between the RELATIVE 4 and the actor-critic models**

(A), (B) and (C): the graphs represent the model estimate of the context (state) values as a function of trial and the task context (the color scheme is the same used in the main text). Bold lines represent the mean; the shaded areas represent the s.e.m. (D), (E) and (F): the bars represent the final option or policy value estimates. . $G_{75}$ and $G_{25}$: options associated with 75% and 25% percent of winning 0.5€, respectively; $L_{75}$ and $L_{25}$: options associated with 75% and 25% percent of losing 0.5€, respectively. The estimates are generated from individual history of choices and outcomes and subject-specific free parameters. (G): the bars represent the difference in BIC between a model and the ABSOLUTE model (Q-learning). Positive values indicate better fit, negative values worst fit, compared to the ABSOLUTE model. (H): the bars represent the posterior probability of the model given the data and the parameters values (calculated based on the LPP; see supplementary Table 1). The dotted line represents chance level (0.25). Errors bars represent s.e.m.

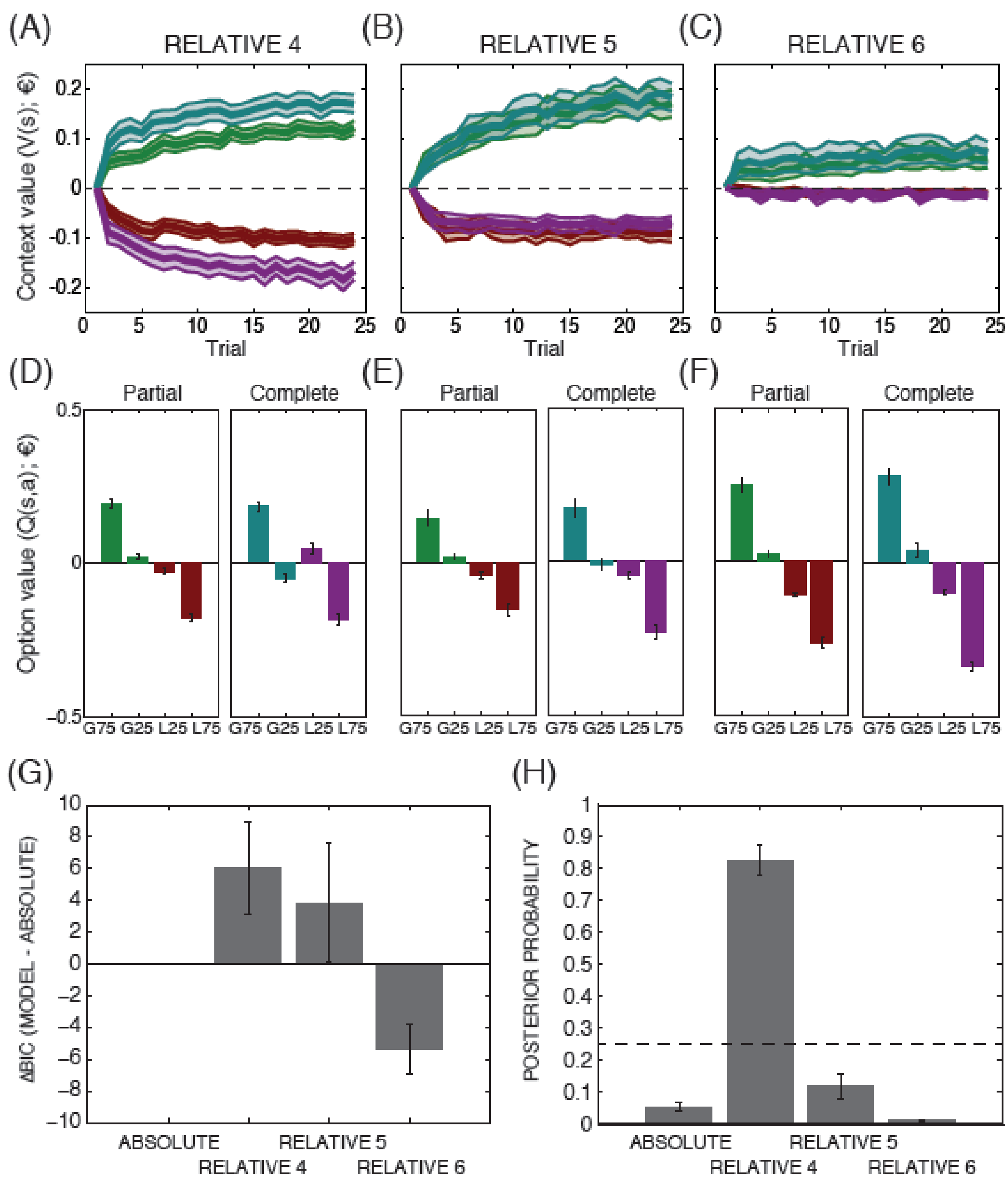


**Supplementary Figure 4: comparison between the RELATIVE 4, 5 and 6 models**

(A), (B) and (C): the graphs represent the model estimate of the context (state) values as a function of trial and the task context (the color scheme is the same used in the main text). Bold lines represent the mean; the shaded areas represent the s.e.m. (D), (E) and (F): the bars represent the final option value estimates. $G_{75}$ and $G_{25}$: options associated with 75% and 25% percent of winning 0.5€, respectively; $L_{75}$ and $L_{25}$: options associated with 75% and 25% percent of losing 0.5€, respectively. The estimates are generated from individual history of choices and outcomes and subject-specific free parameters. (G): the bars represent the difference in BIC between a model and the ABSOLUTE model (Q-learning). Positive values indicate better fit, negative values worst fit, compared to the ABSOLUTE model. (H): the bars represent the posterior probability of the model given the data and the parameters values  (calculated based on the LPP; see supplementary Table 1). The dotted line represents chance level (0.25). Errors bars represent s.e.m.

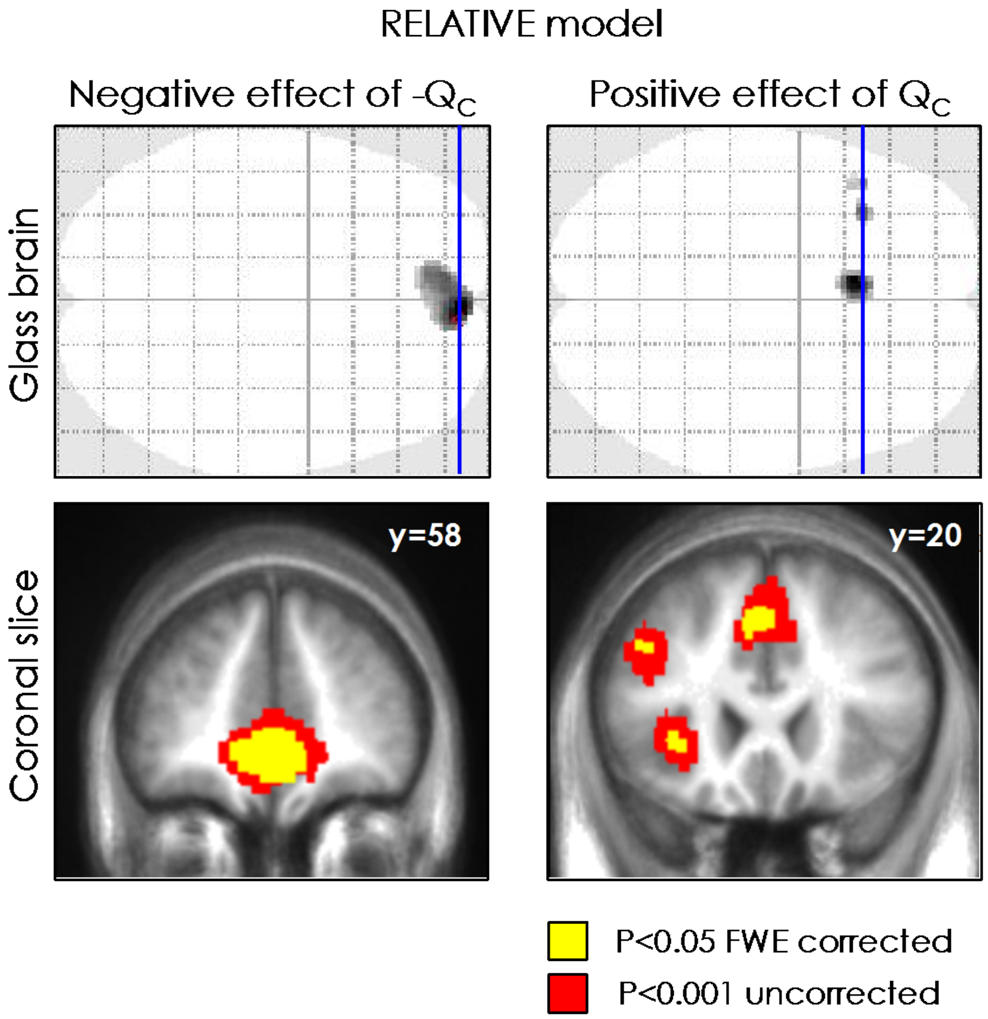


**Supplementary Figure 5: chosen option value representation in the RELATIVE model**

Brain areas correlating positively and negatively with the chosen option value ($Q_C$; left and right column). Significant voxels are displayed on the glass brains (top) and superimposed to slices of the between-subjects averaged anatomical T1 (bottom). Coronal slices correspond to the blue lines on sagittal glass brains. Areas colored in gray-to-black gradient on glass brains and in yellow on slices showed a significant effect at P<0.05, voxel level FWE corrected). Areas colored in red on the slices showed a significant effect at P<0.001, uncorrected. Y coordinates are given in the MNI space. The results are from the GLM using the RELATIVE model parametric modulators (GLM1b).

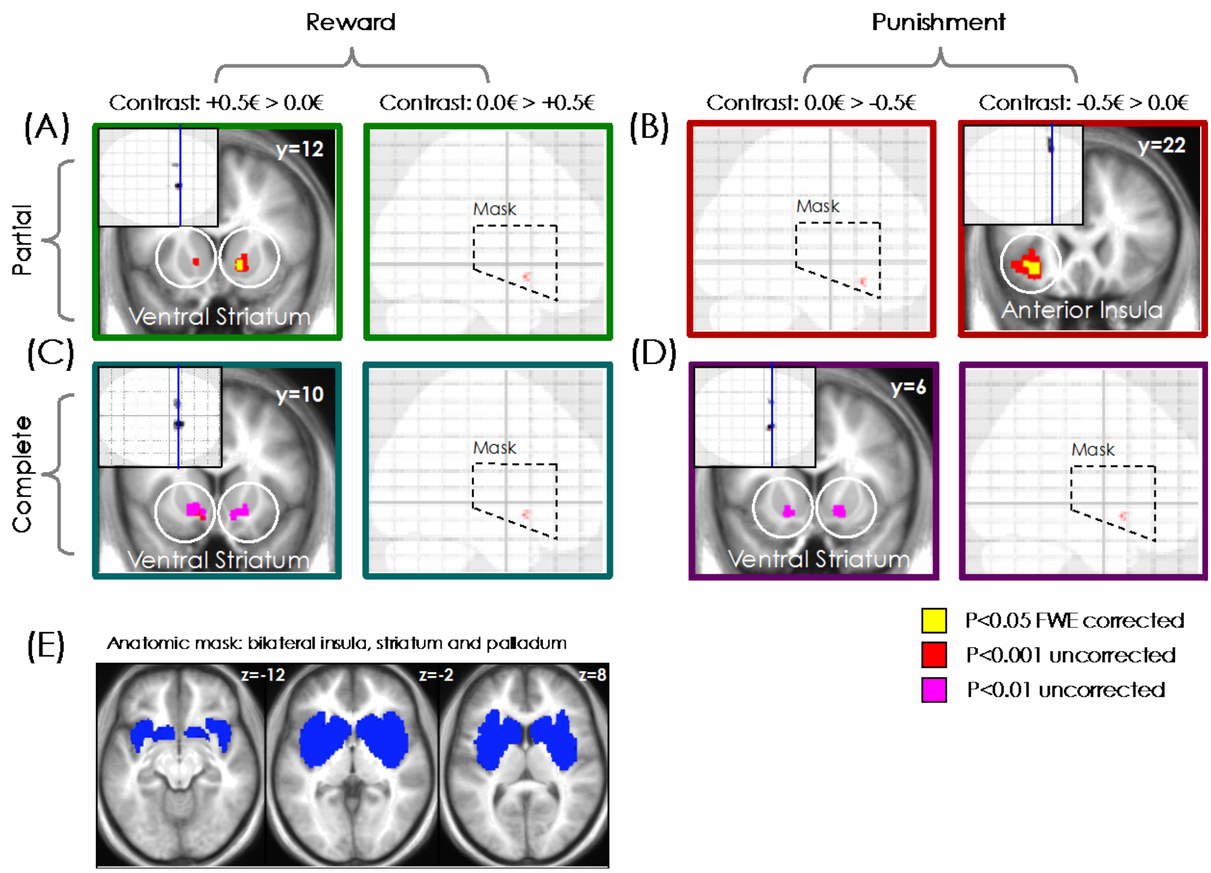


**Supplementary Figure 6: outcome encoding and anatomic mask**

The figure presents the brain activations, concerning the outcome contrasts between the best and the worst outcomes ($R_C$; reward contexts contrast: +0.5€>0.0€; punishment contexts contrast: 0.0€>-0.5€), obtained from the categorical GLM3. Significant voxels are displayed on axial glass brains and superimposed to coronal slices of the between-subjects averaged anatomical T1. Coronal slices correspond to the blue lines on axial glass brains. Y coordinates are given in the MNI space. The results are from the categorical GLM2. Areas colored in gray-to-black gradient on glass brains and in red on slices showed a significant effect (P<0.001, uncorrected in A & B, and P<0.01, uncorrected in C & D). Areas colored in yellow on slices showed a significant effect (P<0.05, FWE mask-level corrected). (A) Significant activations by the best>worst outcome (+0.5€>0.0€) contrast in the reward/partial condition. (B) Significant activations by the best>worst outcome (0.0€>-0.5€) contrast in the punishment/partial condition. (C) Significant activations by the best>worst outcome (+0.5€>0.0€) contrast in the reward/complete condition. (D) Significant activations by the best>worst outcome (0.0€>-0.5€) contrast in the punishment/complete condition. (E) The blue voxels correspond to the anatomic mask used for the study of outcome related activations. The mask includes all voxels classified as striatum, pallidum and insula in the Automatic Anatomic Labeling (AAL) atlas. The mask is superimposed to axial slices of the between-subjects averaged anatomical T1.

**Supplementary Tables**

**Supplementary Table 1: intermediate value cues post learning choice rates**

The table summarizes for both intermediate value cues ($G_{25}$ and $L_{25}$) and feedback information (partial and complete) their experimental and model-derived dependent post-learning choice rate. DATA: experimental data; RELATIVE 4: relative value learning model with delta rule update and context-specific heuristic (best fitting model in all model comparison analyses); ABSOLUTE: absolute value learning model (Q-learning). Data are expressed as mean ± s.e.m. *P<0.05 t-test, comparing the model-derived values to the actual data after correcting for multiple comparisons.

| **Comparison** | **DATA** | **ABSOLUTE** | **RELATIVE 4** |
|---|---|---|---|
| G25 vs G75 (partial) | 0.25±0.07 | 0.11±0.02 | 0.14±0.02 |
| G25 vs L25 (partial) | 0.54±0.08 | 0.84±0.02* | 0.61±0.03 |
| G25 vs L75 (partial) | 0.69±0.07 | 0.95±0.01* | 0.91±0.02 |
| G25 vs G75 (complete) | 0.07±0.03 | 0.09±0.02 | 0.08±0.02 |
| G25 vs L25 (complete) | 0.36±0.08 | 0.88±0.02* | 0.33±0.06 |
| G25 vs L75 (complete) | 0.71±0.07 | 0.97±0.01* | 0.73±0.04 |
| L25 vs G75 (partial) | 0.20±0.06 | 0.04±0.01 | 0.09±0.02 |
| L25 vs G25 (partial) | 0.45±0.08 | 0.16±0.02* | 0.38±0.03 |
| L25 vs L75 (partial) | 0.80±0.05 | 0.83±0.02 | 0.86±0.02 |
| L25 vs G75 (complete) | 0.23±0.06 | 0.03±0.02* | 0.29±0.05 |
| L25 vs G25 (complete) | 0.64±0.08 | 0.11±0.02 | 0.67±0.06 |
| L25 vs L75 (complete) | 0.95±0.04 | 0.90±0.02 | 0.92±0.02 |

**Supplementary Table 2: model comparison of different algorithmic specifications of the RELATIVE model**

The table summarizes for each model its fitting performances. DF: degrees of freedom; LLmax: maximal Log Likelihood; AIC: Akaike Information Criterion (computed with LLmax); BIC: Bayesian Information Criterion (computed with LLmax); LPP: Log of Posterior Probability; XP: exceedance probability (computed from LPP). PP: posterior probability of the model given the data. RELATIVE 4 is the model described in the main text.

| **Model** | **Update** | **Heuristic** | **DF** | **-2*LLmax** | **2*AIC** | **BIC** | **-2*LPP** | **PP** | **XP** |
|---|---|---|---|---|---|---|---|---|---|
| ABSOLUTE | - | - | 3 | 307±20 | 319±20 | 325±20 | 314±20 | 0.08±0.04 | 0.0 |
| RELATIVE 1 | Frequentist | Aspecific | 3 | 306±22 | 318±22 | 324±22 | 315±21 | 0.00±0.01 | 0.0 |
| RELATIVE 2 | Frequentist | Specific | 3 | 303±22 | 315±22 | 322±22 | 313±22 | 0.06±0.01 | 0.0 |
| RELATIVE 3 | Delta rule | Aspecific | 4 | 298±22 | 315±22 | 323±21 | 307±21 | 0.02±0.03 | 0.0 |
| **RELATIVE 4** | **Delta rule** | **Specific** | **4** | **295±22** | **311±22** | **319±22** | **304±21** | **0.84±0.05** | **1.0** |

**Supplementary Table 3: model comparison involving the actor-critic models**

The table summarizes for each model its fitting performances. DF: degrees of freedom (number of free parameters). LLmax: maximal Log Likelihood; AIC: Akaike Information Criterion (computed with LLmax); BIC: Bayesian

Information Criterion (computed with LLmax); LPP: Log of Posterior Probability; XP: exceedance probability (computed from LPP). PP: posterior probability of the model given the data (computed from LPP).

| Model | DF | 2*LLmax | 2*AIC | BIC | 2*LPP | PP | XP |
|---|---|---|---|---|---|---|---|
| ABSOLUTE | 3 | 307±20 | 319±20 | 324±20 | 314±20 | 0.07±0.02 | 0.00 |
| RELATIVE 4 | 4 | 295±22 | 311±22 | 319±22 | 304±21 | 0.51±0.07 | 0.90 |
| ACTOR-CRITIC1 | 4 | 307±22 | 323±22 | 331±22 | 310±22 | 0.22±0.04 | 0.09 |
| ACTOR-CRITIC2 | 4 | 306±22 | 322±22 | 329±22 | 310±22 | 0.19±0.05 | 0.01 |

**Supplementary Table 4: model comparison as a function of different ways to calculate the context value prediction error in the RELATIVE model (i.e. "random-policy", "on-policy", "best-policy").**

The table summarizes for each model its fitting performances. Partial $R_V$: calculation of the context-level outcome term used to update the context value V(s) in the partial feedback conditions. Complete $R_V$: calculation of context-level outcome term used to update the context value V(s) in the complete feedback conditions. LLmax: maximal Log Likelihood; AIC: Akaike Information Criterion (computed with LLmax); BIC: Bayesian Information Criterion (computed with LLmax); LPP: Log of Posterior Probability; XP: exceedance probability (computed from LPP). PP: posterior probability of the model given the data (computed from LPP).

| Model | Partial $R_V$ | Complete $R_V$ | 2*LLmax | 2*AIC | BIC | 2*LPP | PP | XP |
|---|---|---|---|---|---|---|---|---|
| ABSOLUTE | - | - | 307±20 | 319±20 | 324±20 | 314±20 | 0.05±0.02 | 0.00 |
| RELATIVE 4 | $(R_C+Q(s,u))/2$ | $(R_C+R_U)/2$ | 295±22 | 311±22 | 319±22 | 304±21 | 0.82±0.05 | 1.00 |
| RELATIVE 5 | $R_C$ | $R_C$ | 297±22 | 313±22 | 321±22 | 308±21 | 0.11±0.04 | 0.00 |
| RELATIVE 6 | $max(R_C,Q(s,u))$ | $max(R_C,R_U)$ | 306±20 | 322±20 | 330±20 | 315±20 | 0.01±0.01 | 0.00 |

**Supplementary Note 1: behavior**

**I) Reaction times**

*Reaction time analysis provides evidence of relative value encoding.* We also analyzed the reaction times, with the same statistical model used for correct choice rate, and we observed a significant effect of outcome valence ($F=78.0$, $P<0.001$), a marginally statistical effect of feedback information ($F=3.2$, $P=0.09$) and interaction between the two ($F=3.8$, $P=0.06$) (Supplementary Figure 1). Post-hoc test revealed that subjects were slower in the punishment avoidance contexts compared to the reward ones (partial and complete contexts: $T>4.0$, $P<0.001$), whereas the effect of feedback information reached statistical significance only in the punishment context ($T=2.5$, $P<0.05$), but not in the reward one ($T=0.3$, $P>0.5$). Conditioning (Pavlovian-to-instrumental transfer or PIT) as well as decision field theories established a link between chosen option value and reaction times. More precisely they predict that the subjects would take more time if choices are likely to result in negative outcomes[1–3]. We indeed observed this effect (main effect of valence), since subjects were slower when choices potentially led to negative outcomes. However, the trend toward a significant valence x information interaction (driven by faster responses in the punishment/complete context compared to the punishment/partial context) suggests that the reaction times' pattern could not be fully explained by considering option value on an absolute scale. . Importantly, the absence of difference in reaction times in the two reward contexts further indicates that observed pattern could not be explained assuming reaction times a simple function of to the correct response rate that is much higher in the reward/complete contexts compared to the reward/partial. Thus, learning and post-learning results suggest that the observed interaction may derive from relative value encoding: punishment-induced reaction times slowing in the punishment/complete context is smaller compared to the punishment/partial context, as if the option value was less negative as a result of the value contextualization process.

**II) Post-learning test detailed analysis**

*Value inversion in the post-learning test is robust across all possible binary comparisons and confirms relative value encoding.* In the main text we reported post learning choice rate in an aggregate manner, i.e. reporting the probability of choosing an option, taking into account all possible comparisons. The advantage of using this aggregate measure resides in that it is directly proportional to the underlying option value, to which it can be therefore easily compared (see Figure 2B and Figure 3C and 3D). Here we report the results of all possible comparisons involving the intermediate value options (i.e. $G_{25}$, the incorrect option in the reward contexts and $L_{25}$, the correct option in the punishment contexts) (Supplementary Figure 2). The reason to focus on these options is that the ABSOLUTE and RELATIVE models crucially diverge with respect to their post-learning choice rate prediction about $G_{25}$ and $L_{25}$. We analyzed the post learning choice with a three-way ANOVA analysis including option (two levels: $G_{25}$ or $L_{25}$), feedback information (two levels: partial versus complete) and absolute expected value (EV; Probability(outcome) * Magnitude(outcome)) difference between the two options (three levels: low, mid and high) as factors. Crucially, the ABSOLUTE model predicts a main effect of cue ($F=716.3$, $P<0.001$), reflecting higher choice rate for the $G_{25}$, compared to the $L_{25}$ option. The ABSOLUTE model also predicts no significant option x information interaction ($F=0.4$, $P>0.5$), indicating that increased choice rate for the $G_{25}$ compared to the $L_{25}$ was similar in both feedback information conditions, and significant option x EV difference interaction ($F=217.2$, $P<0.001$), reflecting a non-linear increase of post-learning choice rate as a function of EV difference. Importantly, the RELATIVE model predicts a completely different pattern, with no main effect of option (i.e. similar choice rate for the $G_{25}$ and the $L_{75}$ options; $F=1.9$, $P>0.1$), a significant option x information interaction (i.e. an option-specific effect of feedback information on post-

learning choice rate, with higher choice rate for the $L_{25}$ in the complete feedback information; F=51.7, P<0.001), and no significant option x EV difference interaction (i.e. a linear increase of post-learning choice rate as a function of EV difference; F=0.2, P>0.8). Actual post-learning choices systematically fulfilled the predictions of the RELATIVE model, by displaying no significant effect of option (F=3.0, P>0.09), a significant option x information interaction (F=5.1, P<0.05) and no significant option x EV difference (F=0.0, P>0.9). Accordingly, direct systematic comparisons between the actual and the model predicted data, confirmed that only the ABSOLUTE model suffers from significantly diverging from subjects' post-learning behavior (see supplementary Figure 2 and supplementary Table 1).

### III) Post-experiment debriefing

*Post-scanning structured interview fails to reveal acquired explicit knowledge of task factors.* A post-scanning structured interview was administrated to a subgroup of subjects (17/28; 60.7%). The interview was aimed to assess subjects' explicit knowledge of the learning task's features and contingencies. More precisely the structured interview assessed: i) whether or not the subjects were aware about the cues being presented in fixed pairs (choice contexts); ii) how many choice contexts they believed were simultaneously present in a learning session; iii) if they believed or not that rewards and punishments were being separated across choice contexts; iv) if they believed or not that partial and complete feedbacks were being separated across choice contexts. Subjects, on average, correctly retrieved that during learning the cues were presented in fixed choice contexts during learning (correct responses: 88.2%; P<0.001). When asked about how many pairs of cues were presented in a session, subjects, on average, answered 4.6±0.2%, slightly overestimating the correct number (i.e. 4; T=2.2, P<0.05). The task's factors (outcome valence and feedback information) were not significantly reported as discrete, mutually exclusive, features of the choice contexts. Indeed, subjects did not correctly report rewards and punishments as choice context-specific (correct responses: 35.3%; P>0.05). Similarly, subjects did not correctly report partial and complete feedbacks as choice context-specific (correct responses: 47.1%; P>0.2). Thus, as far as explicit knowledge of the task structure can be inferred by the post-scanning structured interview, whereas the existence of discrete choice contexts (states) and their number seemed explicitly grasped by the subjects, the separation between reward and punishment, as well as between partial and complete feedback, conditions, remained implicit. These two features are taken into account by our computational models that i) assume the perception of discrete states (*s*) but ii) treat option and context values as continuous ("model-free") variables instead of categorical ("rule or model-based") ones.

**Supplementary Note 2: computational modeling**

*Model comparison- based justification of the algorithmic specification of the RELATIVE model reported in the main text.* Four different variants of the RELATIVE model were considered, in order to select amongst different possible algorithmic implementations, such as different ways to update the state value (frequentist versus delta rule) and the heuristic employed to obviate the absence of counterfactual outcome ($R_U$) in the partial feedback contexts, when calculating the context-level outcome $R_V$. The first computational question is the learning rule used for context value update. In fact, whereas there is now strong and cumulative evidence that option values are learnt via delta rules[4] it could be that context value updates follow different learning rules. We included RELATIVE models 1 and 2 implementing frequentist inference:

$V_{t+1}(s) = ((t-1)/t)*V_t(s) + (1/t)*R_{V,t}$,

where t is the number of trials and $R_V$ is the context-level outcome at trial t: a global measure that encompasses both the chosen and unchosen options. Frequentist inference is appropriate for environments with no volatility and instantiates a progressive reduction of the learning rate, since new experiences have less weight as the number of trials increases. RELATIVE models 1 and 2 with frequentist update of context value could be advantaged by the fact that they do not require additional free parameters, compared to the ABSOLUTE model. However, for the same reason, they cannot account for interindividual variability. We also included RELATIVE models 3 and 4 implementing the delta rule, which, for analogy with the frequentist update, can be written as:

$V_{t+1}(s) = (1- \alpha_3)*V_t(s) + \alpha_3*R_{V,t}$,

Where $\alpha_3$ is the context value learning rate. Delta rule is appropriate for environments with unknown volatility. RELATIVE models 3 and 4 with delta rule update of context value could be disadvantaged by the fact that they require an additional free parameter ($\alpha_3$), compared to the ABSOLUTE model. However, for the same reason, they can account for interindividual variability. The second computational question concerned the definition of $R_V$. In fact, whereas average outcome trial can be straightforwardly calculated in the complete feedback contexts as the average of the factual and the counterfactual outcomes as follows:

$R_{V,t} = (R_{C,t} + R_{U,t}) / 2$,

the question arises in the partial feedback contexts, where $R_U$ is not explicitly provided. One possibility, (implemented in RELATIVE models 1 and 3) is to replace $R_U$ with $R_M$ (the central – median - task reward: 0.0€), in the partial feedback contexts,:

$R_{V,t} = (R_{C,t} + R_{M,t}) / 2$,

which we define as a "context-aspecific heuristic", in which, simplifying, $R_V = R_{C,t} / 2$, . However, given that $R_V$ is meant to be a context-level measure, in order to incorporate unchosen option information in $R_V$ also in the partial feedback contexts, a possibility, implemented in RELATIVE models 2 and 4, is to consider $Q_t(s,u)$ a proxy of $R_{U,t}$ and calculate $R_{V,t}$ as follows:

$R_{V,t} = (R_{C,t} + Q_t(s,u)) / 2$,

which we define as a "context-specific heuristic".

To sum up, this model space included 5 models. The ABSOLUTE model (Q-learning) and four RELATIVE models which differed in 1) context value update rule ("frequentist" versus "delta rule") and 2) the way $R_V$ was calculated in the partial feedback contexts ("context-aspecific" or "context-specific" heuristic). We submitted these new models to the same parameters optimization procedure and model comparison analyses presented in the main text and involving the Bayesian information criterion (BIC), Akaike information criterio (AIC) and the Laplace approximation

of the model evidence-based calculation of the model posterior probability and exceedance probability[5,6]. Complexity-penalizing model comparison criteria concordantly indicated that the RELATIVE model 4 better accounted for the data (see Supplementary Table 3). Note that priors-independent model comparison criteria (LLmax, AIC and BIC) were smaller (indicating better fit) in all RELATIVE models compared to the ABSOLUTE model, indicating that the finding that relative value learning better accounts for the data was robust across algorithmic variations of the context value update rule. Thus, subsequent analyses in the main text and in supplementary materials have been focused on the comparison RELATIVE model 4 only, to whom we referred simply as "RELATIVE", to stress the main feature of the model instead of its less relevant algorithmic specifications.

*Position of the RELATIVE models within the family of reinforcement learning algorithms: similarity and differences with previous formulations.* The RELATIVE family of models in general, and the RELATIVE 4 model in particular (the best fitting model), computationally embody the ideas behind the two-factor theory that, in simple terms, states that the instrumental action-induced punishment avoidance (cessation of fear in the original formulation) should acquire a positive reinforcement value, in order to sustain instrumental responding, in absence of further negative reinforcement (i.e. successful avoidance)[7]. The RELATIVE models capture this basic intuition of the two-factor theory assuming that, in the punishment conditions, neutral outcomes are computed relative to the negative context values (or state values as they are more frequently called in the reinforcement learning literature). The idea of computationally capturing elements of the two-factor theory by assuming some form of relative value learning has been also proposed in previous computational studies[8,9]. These studies were based on actor-critic or advantage learning models[10,11], and the models proved useful to account for classical avoidance learning results, such as the conditioned avoidance response (CAR) induced via discriminated avoidance procedure. The computational model tested here is inspired by these formulations, with whom it shares the notion of a separate track of action values (i.e. option values Q(s,a)) or policy value (i.e. 'policy' P(s,a)) and state values (V(s)), as well as the calculation of policy values relative to the state value. As a matter of fact the algorithmic implementation of our model only marginally differs from those of these previous models. Thus, in order to justify the introduction of the new model, we run supplementary model comparison analyses. In a first model comparison analysis we compared the RELATIVE 4 model with the actor-critic model, since the latter been explicitly proposed as an effective solution for punishment avoidance learning. Another algorithmic specificity of the RELATIVE 4 model is that V(s) is calculated in an random-policy manner (i.e. it depends on $R_C$ and $R_U$ in the complete feedback contexts and on $R_C$ and Q(s,u) in the partial feedback contexts), as opposite to previous model in which it is calculated on-policy. Thus in the second model comparison analyses presented below, we addressed these issues by (I) comparing the RELATIVE 4 models with two variants of the actor-critic model, and (II) with two variants of the RELATIVE 4 model calculating V(s) based on the current or best policy instead of doing this in an random-policy manner.

### I) Comparison with two variants of the actor-critic model

We compared the RELATIVE 4 model with two variants of the actor-critic model. At each trial t the model calculated a chosen policy prediction error defined as:

$$\delta_{C,t} = R_{C,t} - V_t(s),$$

where V(s) is the value of the current choice context s and $R_C$ is the outcome of the chosen policy (factual outcome). This prediction error is then used to update the chosen policy value (P(s,c)) using a delta-rule:

$$P_{t+1}(s,c) = P_t(s,c) + \alpha_1 \delta_{C,t},$$

where $\alpha_1$ is the learning rate for the chosen option. We extended the actor-critic model in order to integrate counterfactual learning, as we have done for the other models. Thus, in the complete feedback contexts, the model also calculates an unchosen policy prediction error:

$\delta_{U,t} = R_{U,t} - V_t(s)$,

where $R_U$ is the outcome of the unchosen policy (counterfactual outcome). This prediction error is then used to update the unchosen policy value (P(s,u)) using a delta-rule:

$P_{t+1}(s,u) = P_t(s,u) + \alpha_2 \delta_{U,t}$,

where $\alpha_2$ is the learning rate for the unchosen option. The two variants of the actor critic model differ in the way the context value V(s) is then updated. In the first, more "classical", variant (ACTOR-CRITIC 1) the chosen policy prediction error is also used to update the context value in all choice contexts:

$V_{t+1}(s) = V_t(s) + \alpha_3 \delta_{C,t}$,

where $\alpha_3$ is the learning rate for the context value. In a second variant (ACTOR-CRITIC 2) the context value update also takes into account the unchosen policy prediction error:

$V_{t+1}(s) = V_t(s) + \alpha_3 \delta_{C,t} + \alpha_3 \delta_{U,t}$.

We submitted these new models to the same parameters optimization procedure and model comparison analyses presented in the main text and involving the Bayesian information criterion (BIC), Akaike information criterio (AIC) and the Laplace approximation of the model evidence-based calculation of the model posterior probability and exceedance probability[5,6]. The model space included the ABSOLUTE model as a reference point and the RELATIVE 4 (the best fitting model of the main model comparison). Including the choice temperature, the ACTOR-CRITIC models 1 and 2 have four free parameters, as the RELATIVE 4 model has. The results (see Supplementary Table 3 and Supplementary Figure 3) indicated that the RELATIVE 4 model provides a better account of the data, compared to both the actor-critic models.

**II) Comparison with different ways to calculate the context value calculation**

We also devised two additional variants of the RELATIVE models. These variants assume the context value being calculated based on the current (RELATIVE 5) or the best (RELATIVE 6) policy. More specifically, these models essentially differ from the RELATIVE 4 in the way they calculate the $R_{V,t}$: the context-level outcome at trial t, which is used to update the context, value V(s). In the RELATIVE 4 model $R_V$ was calculated based on the $R_C$ and Q(s,u), in the partial feedback contexts, and based on $R_C$ and $R_U$, in the complete feedback contexts (i.e. "random-policy" since independent from the subjects' choice). This choice was motivated by conceiving V(s) as a reference point as much neutral as possible in respect to the current obtained outcomes, supposing that the subjects do take all feedback into account (thus being random-policy) to estimate the context value (see "Conclusions on supplementary computational analyses"). However this choice is not frequent in the current panorama of reinforcement learning algorithms. In the RELATIVE 5 model for all choice contexts the context level outcome is defined as:

$R_{V,t} = R_{C,t}$ .

The context value V(s) is therefore calculated considering the ongoing policy ("on-policy"). This is the most frequent way to calculate the context value in the reinforcement learning literature. Note that the RELATIVE 5 is analogous to the advantage learning algorithm extended to also, once included the counterfactual learning module[10]. Another tempting possibility, particularly relevant in presence of complete feedback information, is to calculate the context

value based on the best policy. The RELATIVE 6 model implements this possibility, in fact in the partial information choice contexts the context level outcome is defined at:

$R_{V,t} = max(R_C, Q(s,u))$,

whereas in the complete information choice contexts it is defined as:

$R_{V,t} = max(R_C, R_U)$.

We submitted these new models to the same parameters optimization procedure and model comparison analyses presented in the main text. The model space included the ABSOLUTE model, as a reference point, and the RELATIVE 4, 5 and 6 models. The RELATIVE 5 and 6 models have four free parameters, as the RELATIVE 4 model has. The results (see Supplementary Table 4 and Supplementary Figure 4) indicated that the RELATIVE 4 model provides a better account of the data, compared to RELATIVE models 5 and 6, thus supporting the random-policy calculation of the context value in this task. Another interesting metric to evaluate in each model the gain of implementing relative value learning is to look at the values of the context learning rate $\alpha_3$. In fact, when $\alpha_3 = 0$ the RELATIVE models reduce to the ABSOLUTE model. In the RELATIVE model 4 only 4 subjects (14%) were fitted with $\alpha_3=0$. This percentage slightly increased in the RELATIVE model 5 (N=7; 25%) and dramatically increased in the RELATIVE model 6 (N=17; 61%), further confirming the relatively poor fitting performances of their context value update scheme (this analysis was performed on the parameters retrieved with likelihood maximization).

### III) Conclusions on supplementary computational analyses

Whereas previous computational studies suggested that the actor-critic architecture could provide a good explanation for conditioned avoidance response[8,9], we found that in our task the RELATIVE 4 outperformed the actor-critic models. One important difference compared to the actor critic model is that the RELATIVE 4 model can be reduced to Q-learning assuming the contextual learning rate ($\alpha_3=0$), whereas the actor critic cannot. This lack of flexibility may at least partly explain the overall poor group-level performances. We also note the important differences between the discriminate avoidance procedure and our paradigm. In the former the contingencies are deterministic, avoidance learning is studied in isolation and the "avoidance learning paradox" consists in the long lasting insensitivity to extinction of the conditioned responses, despite the absence of further reinforcement. In our paradigm, the contingencies are probabilistic (thus with overlapping outcomes from the correct and incorrect choices), avoidance learning is not studied in isolation, but in opposition to reward seeking behavior and the "avoidance learning paradox" consists in similar performance in the reward punishment domain, despite the fact that the performance-induced sampling bias would predict enhanced performances in the reward domain. These important differences should be also taken into account, when interpreting the relatively poor performances of the actor-critic models in our task. On the other side the good potential of the actor-critic model to explain the post-test results (Supplementary Figure 3E and 3F), further illustrates the conceptual proximity between this influential algorithm and the RELATIVE model 4.

We also found that "random-policy" calculation of the context value in the RELATIVE model 4 provided a better explanation of instrumental choices compared to other forms of context value calculations (on-policy or best-policy) Interestingly, the RELATIVE models 5 and 6 also failed to capture the value inversion of the intermediate value cues in the complete feedback conditions (see Supplementary Figure 4E-F). As a matter of fact, in most of the classical instrumental conditioning (and machine learning) paradigms the agents are presented to only one type of choice context (either reward or punishment, as in the discriminated avoidance task, and there is almost no example

of complete feedback information[1])[11,12]. Thus, in presence of only one type of choice context, the model predictions obtained using on-policy or random-policy context value (V(s)), can hardly diverge. In such mono-dimensional tasks, on- and random-policy context values would display a similar trend across trials and the eventual differences in their magnitude can easily be neutralized by rescaling parameters, such as coefficients or learning rates (see Supplementary Figure 4A-C). We believe that we were precisely able to rule out on-policy (and best-policy) context value, thanks to the presence of multiple, different choice contexts in our design. In particular, both the simultaneous contrasts between reward and punishment and between partial and complete feedback information contributed to highlight this feature of the best fitting model. In fact, only the random-policy context values i) were symmetrical in respect to the valence, thus permitting similar performances in the reward and punishment domains, and ii) were enhanced in magnitude in the complete feedback contexts, thus permitting the value inversion of the intermediate value cues in the complete feedback conditions (see Supplementary Figure 3A-F). Furthermore, the importance of being on-policy has been mainly stressed in problems with a risk of substantial/lethal punishments such as the cliff simulation where random-policy algorithms such as Q-learning cannot avoid sometimes falling in the cliff due to occasional exploratory decisions[11,12]. In our case, it is reasonable to consider that human subjects do not fear being harmed when interacting with the screen. In fact, we believe that this algorithmic difference between the standard view of the context value V(s) (on-policy) and ours (random-policy) betrays a more profound difference concerning the psychological intuitions behind these quantities. Whereas in most reinforcement learning models V(s) is conceived as a "Pavlovian" anticipation of the reward (or punishment) to come, aimed to elicit automatic motor effects[2,3], in our model it represents a more abstract signal, subserving value contextualization for efficient encoding purposes[13–15]. In the light of these interpretation it is easy to understand why in the framework of a "motor preparation", the context value needs to be calculated in an on-policy manner (preparation to an outcome), whereas in the framework of a "efficient coding", the context value has to be calculated in a random-policy manner. In principle both quantities (on- and off- policy context values) could exist in the brain and express their effects in different behavioral measures. Further work, probably implicating a deeper analysis of reaction times (a good candidate for Pavlovian effects) could shed light on this topic. Finally, we are not without acknowledging that an random-policy calculation of context value could rapidly become computationally challenging in learning situations implicating more than two options. Further studies are needed to uncover the learning heuristics implemented in such cases.

[1]This is less true in behavioral economics literature, where counterfactual (or "fictive") learning takes a more important place.

**Supplementary Note 3: written task instructions**

The subject read the learning test instructions before the training session, outside the scanner. The experimenter read the post-learning instructions to the subject, while he/she was in the scanner, after the last (fourth) functional acquisition, before starting the T1 anatomic acquisition.

### Learning test instructions

The experiment is divided in four sessions, of about 12 minutes each. There will be two training sessions (a longer one outside and a shorter one inside the scanner) before the starting of the fMRI experiment.

You are asked to choose in each round one of two abstract symbols. The symbols will appear on the screen to the left or the right of a fixation cross. To choose one of the two symbols you should press the right or left button. After few seconds a cursor will appear under the chosen symbol confirming your choice. If you do not press any button, the cursor will appear at the center of the screen, and your result will be disadvantageous.

As an outcome of your choice you may:

-gain 50 cents (+0.5€)
-get nothing (0€)
-loose 50 cents (-0.5€)

The outcome of your choice will appear on the top of the chosen symbol, and will be always indicated by the position of the cursor. The two symbols are not equivalent (identical). One of the two symbols is on average more advantageous or less disadvantageous, in the sense that it makes you winning more often or loosing less often than the other. The goal of the experiment is to gain as much as you can.

In some trials the information about the outcome of the unchosen option will be also provided. Note that your earnings will correspond only to the chosen option.  At the end of each session the experimenter will communicate your earnings for that session. Your final earnings will correspond to the sum of the earnings of the four sessions.

### Post-learning test instructions

The test will last 5 minutes with no training.

The goal of the next test is to indicate the symbol with the higher value from the last (fourth) session. At any trial, you are asked to choose between two symbols pressing the corresponding button. Your choice will be immediately recorded and will be confirmed with the presence of a cursor that will appear under the chosen stimulus.

It will not always be the case that the shown symbols would have been presented together in the previous session. Please try to give an answer even if you are not completely sure.

**Supplementary references**


1. Busemeyer, J. R. & Townsend, J. T. Decision Field Theory: A Dynamic-Cognitive Approach to Decision Maling in an Uncertain Environment. *Psychol Rev* **100,** 432–459 (1993).

2. Niv, Y., Joel, D. & Dayan, P. A normative perspective on motivation. *Trends Cogn Sci* **10,** 375–81 (2006).

3. Guitart-Masip, M., Duzel, E., Dolan, R. & Dayan, P. Action versus valence in decision making. *Trends Cogn Sci* **18,** 194–202 (2014).

4. Skvortsova, V., Palminteri, S. & Pessiglione, M. Learning To Minimize Efforts versus Maximizing Rewards: Computational Principles and Neural Correlates. *J Neurosci* 1–11 doi:10.1523/JNEUROSCI.1350-14.2014

5. Daunizeau, J., Adam, V. & Rigoux, L. VBA: a probabilistic treatment of nonlinear models for neurobiological and behavioural data. *PLoS Comput Biol* **10,** e1003441 (2014).

6. Khamassi, M., Quilodran, R., Enel, P., Dominey, P. F. & Procyk, E. Behavioral Regulation and the Modulation of Information Coding in the Lateral Prefrontal and Cingulate Cortex. *Cereb Cortex* bhu114– (2014). doi:10.1093/cercor/bhu114

7. Mowrer, O. H. *Learning theory and behavior.* (John Wiley & Sons Inc, 1960). doi:10.1037/10802-000

8. Moutoussis, M., Bentall, R. P., Williams, J. & Dayan, P. A temporal difference account of avoidance learning. *Network* **19,** 137–60 (2008).

9. Maia, T. V. Two-factor theory, the actor-critic model, and conditioned avoidance. *Learn Behav* **38,** 50–67 (2010).

10. Baird, L. C. Reinforcement learning in continuous time: advantage updating. in *Proc 1994 IEEE Int Conf Neural Networks* **4,** 2448–2453 (IEEE, 1994).

11. Sutton, R. S. R. S. & Barto, A. G. A. G. *Reinforcement Learning: An Introduction. IEEE Trans Neural Networks* **9,** (MIT Press, 1998).

12. Dayan, P. & Abbott, L. F. *Theoretical Neuroscience: Computational and Mathematical Modeling of Neural Systems (Computational Neuroscience).* (MIT Press, 2005). at <http://www.gatsby.ucl.ac.uk/~dayan/book/>

13. Louie, K. & Glimcher, P. W. Efficient coding and the neural representation of value. *Ann N Y Acad Sci* **1251,** 13–32 (2012).

14. Padoa-schioppa, C. & Rustichini, A. Rational Attention and Adaptive Coding : *Am Econ Rev Pap Proc* **104,** 507–513 (2014).

15. Rangel, A. & Clithero, J. a. Value normalization in decision making: theory and evidence. *Curr Opin Neurobiol* **22,** 970–81 (2012).
